# Clinical risk score for cardiac death or heart failure hospitalization in moderate aortic stenosis

**DOI:** 10.1186/s44156-026-00110-w

**Published:** 2026-03-16

**Authors:** Jonathan Sen, Agus Salim, Dulari Hakamuwa Lekamlage, Sudhir Wahi, Thomas H. Marwick

**Affiliations:** 1 Baker Heart and Diabetes Institute, 75 Commercial Road, Melbourne, VIC 3004 Australia; 2 Princess Alexandra Hospital, Brisbane, QLD Australia; 3 Department of Cardiometabolic Health, University of Melbourne, Melbourne, Australia; 4 Institute for Mental and Physical Health and Clinical Translation (IMPACT), Deakin University, Geelong, Australia; 5 Western Health, Melbourne, VIC Australia

**Keywords:** Aortic stenosis, Moderate aortic stenosis, Risk prediction

## Abstract

**Background:**

The association between moderate aortic stenosis (AS) and adverse cardiovascular outcomes is heterogeneous. Outcomes are likely dependent on clinical factors but no formal means of integrating these variables has been defined. This study aims to develop and validate a risk score to predict 5-year cardiac mortality or heart failure (HF)-related hospitalization in moderate AS.

**Methods:**

This was a retrospective cohort study that included patients diagnosed with moderate AS. Patients with aortic valve intervention or severe AS were excluded at baseline and censored at follow up. Multivariable Cox proportional hazard model with LASSO penalty followed by a greedy selection algorithm was used to derive a risk score, which was then externally validated for predicting the 5-year composite risk of cardiac mortality or HF-related hospitalization.

**Results:**

The derivation cohort included 2,212 patients with moderate AS (mean age 73.4±11.0 years, 65.7% male) with median follow-up of 4.3 years (interquartile range: 1.7-5). The top 10 variables included in the risk score included 6 echocardiographic variables (left ventricular (LV) end-diastolic diameter, LV outflow tract velocity-time integral, E-wave, end-diastolic left ventricular posterior wall thickness and moderate/severe mitral regurgitation, moderate/severe tricuspid regurgitation) and 4 clinical variables (age, diastolic blood pressure, acute coronary syndrome, hyperlipidemia). The *C*-statistics for the score were 0.70 (95% CI: 0.67–0.76) in the internal validation dataset and 0.75 (95% CI: 0.70–0.79) in the external validation dataset (*n* = 1,141), demonstrating good predictive performance.

**Conclusions:**

This moderate AS risk score, based on demographic and clinical features, as well as conventional echocardiographic parameters, predicts outcomes in patients with moderate AS. This quantification of risk may help with shared decision-making about possible interventions, planning the frequency of follow-up, and selecting candidates for potential randomized trials in this heterogeneous population.

**Graphical Abstract:**

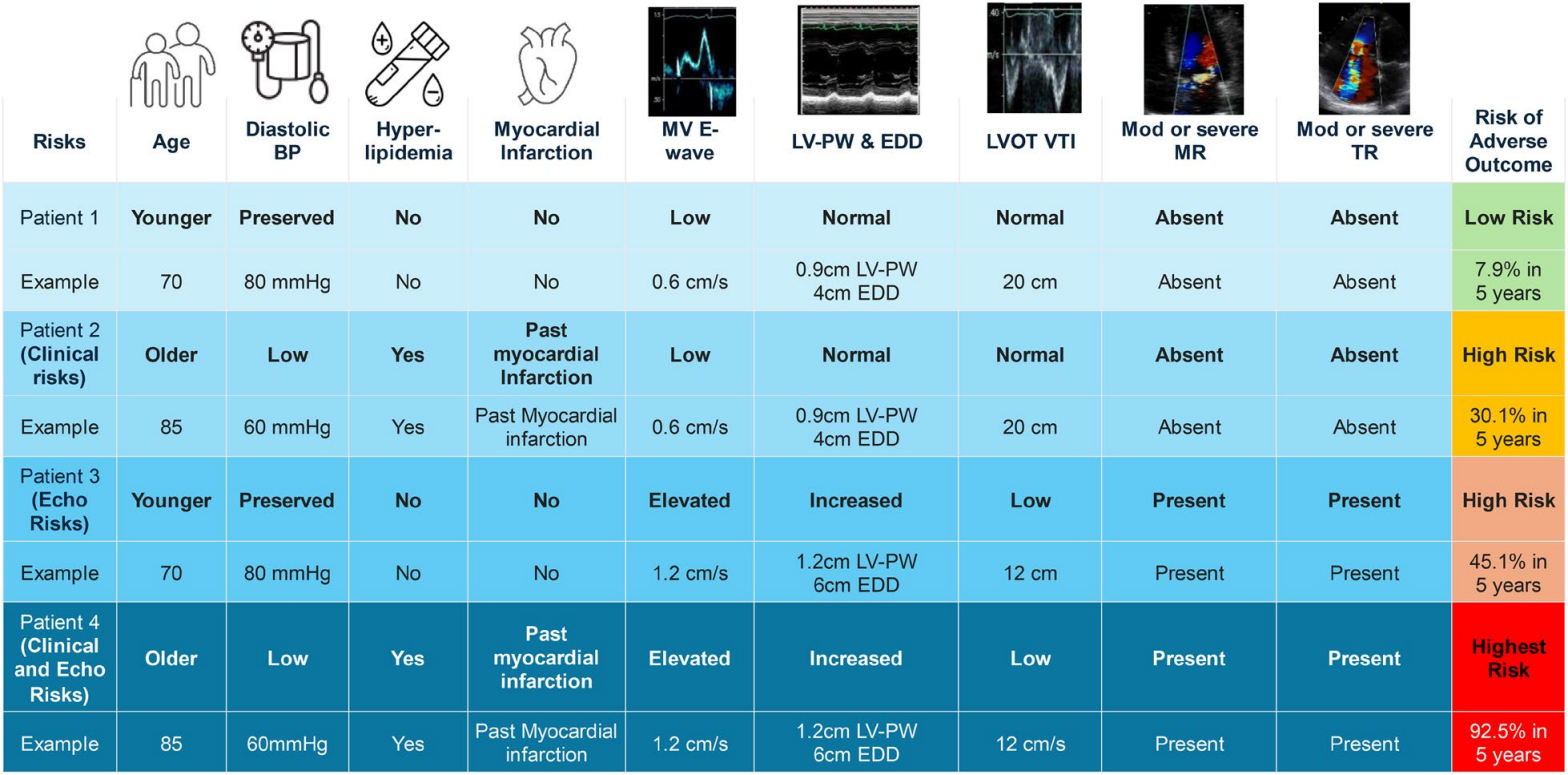

**Supplementary Information:**

The online version contains supplementary material available at 10.1186/s44156-026-00110-w.

## Introduction

Aortic stenosis (AS) is a major and growing public health burden associated with substantial morbidity and mortality, most commonly affecting the elderly population [1]. Although guidelines pertain to severe AS, moderate AS is also associated with risk [2]. A recent systematic review and pooled meta-analysis of 10 studies comparing outcome between moderate AS (11,527 patients) and no/mild AS (398,153 patients) showed 2.5-fold increase in hazard, with 15‐year overall survival rates of 23.3% (95% CI, 19.1%–28.3%) and 58.9% (95% CI, 58.1%–59.7%), respectively [3].

This information causes disquiet among patients with moderate AS and their clinicians. However, while the increment of risk is clear, the drivers of risk (and hence the appropriate management strategy) are more complicated. It is possible that moderate AS itself carries risk, although the mechanism of this is obscure. Patients with moderate AS may develop complications or die from the progression to severe AS. While this process is generally slow (averaging 4.1 mmHg/year) it is also variable (2.80–5.40 mmHg/year) [4], so some patients may progress to severe AS between follow-up visits. However, a perhaps more likely explanation is that cardiovascular risk in moderate AS is driven by non-valvular diseases which are common in this population, including LV dysfunction, hypertension and coronary artery disease, among others. Classification of AS based only on echocardiographic criteria may not adequately capture the heterogeneity of risk drivers in this disease [5, 6]. Risk models based only on echocardiographic parameters omit the frequency and impact of comorbidities in elderly patients with AS [5, 7–10], so predictive models to extend beyond conventional echocardiographic measures [11]. The aim of this study was to combine these clinical features into the assessment of risk, to guide outcomes and treatment responses in moderate AS.

## Methods

### Study design and data linkage

This is a cohort study of prospectively-gathered, but retrospectively-analyzed echocardiographic and clinical information from Metro South Health (Queensland, Australia), used to create a derivation dataset spanning from January 1, 2009, to August 1, 2023. These data were derived from a clinical echocardiographic database, with demographics, clinical, and imaging data from Queensland Cardiac Outcomes Registry (QCOR) [12], which links hospital datasets (cardiac catheterization lab, cardiac surgery, echocardiography, electrophysiology, International Classification of Diseases 10th Edition (ICD-10) codes and death data).

The external validation cohort consisted of patients identified from transthoracic echocardiograms obtained from echocardiographic picture archives at Western Health (Melbourne, Australia) between January 1, 2013, and April 9, 2021. An accredited sonographer with over 20 years of experience independently validated selected echocardiographic parameters with focus on AV [9] in the external cohort only by ensuring only appropriate values were curated and peak gradients acquired by reviewing raw Digital Imaging and Communications in Medicine image data sources [9]. The echocardiographic parameters were provided to Centre for Victorian Data Linkage (CVDL) to carry out linking with prospectively acquired external datasets (hospital admissions, emergency, non-admitted heath, and death dataset from Victoria) between January 1, 2000, to December 31, 2021.

### Study population

Current guidelines have defined moderate AS using the following echocardiographic parameters: aortic valve area (AVA) 1.00-1.49 cm^2^, indexed AVA 0.60–0.85 cm^2^/m^2^, peak velocity 3.00–3.99 m/s, aortic valve (AV) mean pressure gradient (MPG) 25–39 mmHg or dimensionless severity index (DSI) 0.25–0.49. However, the guidelines do not have a strict hierarchy among the echocardiographic parameters used to classify moderate AS, nor do they specify how many criteria must be met to assign the moderate severity category. A highly cited recent paper included patients having any of the velocity, MPG or AVA within the stated range [2], and previous work has used any one of these criteria in isolation. In our cohort, we adopted this “single-criterion” approach for identifying moderate AS in the presence of any one criterion because it aligns with the prevailing practice in contemporary literature and maximizes sensitivity for detecting patients who may benefit from closer surveillance or early intervention. By contrast, we required at least 2 severe-AS criteria (AVA < 1 (or indexed AVA < 0.6 cm^2^/m^2^), peak velocity ≥4 m/s, MPG≥40 mmHg, or DSI < 0.25) to define severe AS and excluded from study. This stricter threshold reflects the higher risk associated with severe AS and mitigates the possibility of over classifying patients with isolated extreme values—such as an isolated peak gradient of 80 mmHg—in the moderate category. Requiring multiple concordant severe parameters improves specificity and reduces misclassification.

To enter the study, patients had to be aged at least 18 years with available demographic, clinical and echocardiographic information. Patients were identified from echocardiogram picture archiving and communication system and included patients who did not undergo AV intervention at baseline. To ensure data integrity and to minimize bias, patients were excluded if all AV echocardiographic parameters were missing (i.e. AVA, peak velocity, MPG and DSI), or satisfied severe AS. There were 21% with at least one missing AV parameter (79% complete AV parameter data) in internal dataset; and 8.4% with at least one missing AV parameter (91.4% complete AV parameter data) in external dataset. Patients were followed from date of first diagnosis of moderate AS to the first event date (heart failure hospitalization or cardiac death) and censored at the first diagnosis of severe AS (as defined above), AV intervention, or up to 5 years.

### Echocardiograms

Transthoracic echocardiograms were re-validated during the study according to the guidelines established by the American Society of Echocardiography [5], regardless of the original acquisition date. The following parameters were measured: the left ventricular (LV) outflow tract (LVOT) diameter, the velocity-time integral (VTI) of the LVOT, the peak velocity of the LVOT, and MPG. The AVA was calculated using the continuity equation with the VTI and DSI is calculated by dividing the velocities of LVOT and AV [13].

### Outcome definitions

The outcome of interest was the composite of cardiac death or heart failure hospitalization. Secondary outcomes included individual components of composite endpoints (cardiac death and heart failure hospitalization), and all-cause mortality (i.e. death attributable to any cause). Outcomes were identified from admitted episode datasets and death registries from QCOR or CVDL. Data was de-identified after data linkage and were not investigator-reported. Established clinical criteria and diagnostic codes were used to ensure standardized and objective measurements across the derivation and validation datasets. Central adjudication was not possible.

Cardiac death was extracted from death registries in QCOR or CVDL by text-mining causes of death such as heart disease, myocardial infarct, coronary artery disease, acute coronary syndrome, cardiopulmonary failure, ischemic heart disease, poor or reduced left ventricular ejection fraction (LVEF). Heart failure hospitalization was defined as one or more hospitalizations from admitted episode datasets from QCOR or CVDL extracted from ICD-10 codes that occurred after the echocardiographic study. The full definitions of each outcome are described in Supplementary Table 1.

### Variables associated with outcomes

A comprehensive set of potential variables was considered for inclusion in the Cox regression model. These variables encompassed demographic information, clinical characteristics, laboratory results, imaging findings, and procedures performed at baseline during first identification of AS based on echocardiogram. Key variables included age, sex, AVA, peak velocity, MPG, LV dimensions, RV systolic function, RV dimensions, LVEF, blood pressure, atrial fibrillation, acute coronary syndrome, diabetes mellitus, hyperlipidemia, chronic kidney disease, other valvular disease, and pacemaker implantation (full list in Supplementary Table 2).

### Statistical analysis

Descriptive statistics were used to summarize demographic, clinical, and echocardiographic parameters of the study population. Continuous variables were reported as mean ± standard deviation, while categorical variables were presented as frequencies and percentages. Outliers of continuous variables that were more than 4 times standard deviation, or clinically meaningful pre-specified threshold for each variable were excluded. Variables with > 20% missing values (except for blood pressures) were excluded (Supplementary Table 3). The missing values in variable data were imputed using an iterative imputer consisting of 1000 iterations and a random forest regressor with 300 trees.

Participant characteristics between the derivation dataset and external validation dataset were compared using Fisher’s exact test (dichotomous categorical variables), Chi-square test (> 2 classes for categorical variables) and Kruskal-Wallis test (non-normally distributed continuous variables), where *p* < 0.05 was considered statistically significant. The model was developed to predict the likelihood of each clinical outcome based on the selected variables.

#### Internal validation

The derivation dataset was divided using a split-data approach (80:20 ratio) into the internal training set (80%) and internal test set (20%). Participant characteristics between the internal validation training and test datasets were assessed in the same manner as between the derivation and external validation datasets.

#### Modeling

A Cox proportional hazard model using a two-step approach, initially with least absolute shrinkage and selection operator (LASSO) for variable selection, was developed to assess the effects of the variables on the risk of composite of cardiac death or heart failure hospitalization. Subsequently, a greedy selection algorithm was used with the variables selected from LASSO with stepwise forward and backward model selection by Akaike Information Criterion. The top ten variables were then selected based on lowest *p* values followed by highest beta coefficients in a refitted final Cox proportional hazards regression model. The effect of selected variables was expressed as hazard ratios (HR) with 95% confidence intervals (CI). Analysis of variance (Wald statistics) was used to assess the significance of the effect on time to events (composite of cardiac death or heart failure hospitalization) from the Cox model.

Predictions were applied to the internal test set and the external validation set. The *C*-statistic was determined at 5 years as well as time-dependent *C*-statistic from 1 to 5 years from censored data using Akritas’ nearest neighbor estimation method were then determined using *survivalROC* package in R [14]. To assess the robustness of the developed risk score, a sensitivity analysis was performed by using LASSO followed by greedy algorithm without minimization to top 10 variables, and re-evaluating the predictive performance of the models.

Both the internal and external validation datasets were split into tertiles with three risk scenarios: low, intermediate, and high risk. Time to event analysis was used to describe the hazard functions of the risk score tertiles and survival curves were compared using the log-rank test. Hazard ratios for the primary and secondary outcomes per risk score tertile were computed using Cox proportional hazards models. For cardiac death and heart failure hospitalization, death from other causes was considered a competing risk in the models. For severe AS, AV intervention and all-cause death were a competing risk. Hazards of the sub-distribution for the outcomes derived from cumulative incidence function were used and corrected for competing risk [15]. A *p*-value < 0.05 was considered statistically significant.

All statistical analyses were performed on R version 4.1.2 [16] using packages Table 1 [17] and *DescTools* [18] to summarize data, *randomForest* package [19] to impute missing data, *survival* [20] to fit proportional hazards regression model, *stepAIC* from *MASS* package [21] to select top variables, *epiR* [22], *pROC* [23], and *survivalROC* packages [24] to evaluate performance of the models, *survminer* [25] to develop cumulative hazard plots, *glmnet* [26] to perform LASSO Cox proportional-hazards models and *cmprsk* [27] to conduct competing risk analysis. A web-based Shiny application was developed to implement the final model.

**Table 1:** Baseline characteristics of moderate aortic stenosis patients in derivation and external validation cohorts

Characteristics	Derivation cohort	External validation cohort	Overall	*P*-value
	(*N* = 2212)	(*N* = 1141)	(*N* = 3353)	
**Demographic**				
Age (years)	73.4 (11.0)	73.9 (13.5)	73.6 (11.9)	0.517
BMI (kg/m^2^)	28.1 (9.80)	28.9 (20.6)	28.4 (14.4)	0.374
Height (cm)	169 (9.90)	163 (11.9)	167 (10.9)	< 0.001
Male, n (%)	1454 (65.7%)	585 (51.3%)	2039 (60.8%)	< 0.001
Weight (kg)	84.0 (19.8)	80.2 (20.6)	82.7 (20.1)	< 0.001
**Echocardiographic**				
AV mean gradient (mmHg)	19.1 (7.43)	13.4 (8.40)	17.1 (8.23)	< 0.001
AV V_max_ (cm/s)	267 (67.2)	231 (73.8)	254 (71.5)	< 0.001
AV VTI (cm)	58.8 (16.4)	48.1 (18.4)	55.2 (17.8)	< 0.001
AVA (cm^2^)	1.43 (0.292)	1.41 (0.317)	1.42 (0.301)	0.242
AVAi (cm^2^/m^2^)	0.742 (0.152)	0.754 (0.182)	0.746 (0.163)	0.128
DSI	0.458 (1.17)	0.464 (0.144)	0.460 (0.956)	0.986
Diastolic BP (mmHg)	73.3 (8.08)	71.0 (7.28)	72.5 (7.89)	< 0.001
Systolic BP (mmHg)	137 (16.1)	132 (12.6)	135 (15.2)	< 0.001
Dilated RV, n (%)	211 (9.5%)	164 (14.4%)	375 (11.2%)	< 0.001
E/A	1.05 (0.452)	1.17 (0.534)	1.09 (0.485)	< 0.001
E/e’	9.05 (6.47)	15.1 (5.46)	11.1 (6.79)	< 0.001
LA Area (cm^2^)	24.4 (5.84)	24.3 (6.48)	24.4 (6.07)	0.819
LA volume (mL)	85.0 (29.9)	83.7 (29.3)	84.6 (29.7)	0.499
LV PW DIAS (cm)	1.04 (0.188)	1.04 (0.177)	1.04 (0.184)	0.448
LV SEPT DIAS (cm)	1.15 (0.218)	1.14 (0.197)	1.15 (0.211)	0.0689
LVEDD (cm)	4.72 (0.709)	4.86 (0.790)	4.77 (0.740)	< 0.001
LVEDV (mL)	113 (35.6)	109 (40.0)	112 (37.2)	0.0239
LVEF (%)	53.9 (13.2)	51.4 (12.5)	53.0 (13.0)	< 0.001
LVMI (g/m^2^)	102 (24.3)	113 (29.0)	106 (26.5)	< 0.001
LVOT diameter (cm)	2.23 (0.207)	2.08 (0.216)	2.18 (0.221)	< 0.001
LVOT VTI (cm)	21.4 (5.25)	20.5 (7.12)	21.1 (5.96)	< 0.001
Moderate or Severe AR, n (%)	74 (3.3%)	41 (3.6%)	115 (3.4%)	0.933
Moderate or Severe MR, n (%)	182 (8.2%)	167 (14.6%)	349 (10.4%)	< 0.001
Moderate or Severe MS, n (%)	16 (0.7%)	11 (1.0%)	27 (0.8%)	0.761
Moderate or Severe TR, n (%)	153 (6.9%)	163 (14.3%)	316 (9.4%)	< 0.001
MV deceleration time (msec)	239 (67.2)	220 (63.7)	232 (66.6)	< 0.001
MV E wave (cm/s)	89.6 (34.0)	97.2 (29.4)	92.2 (32.7)	< 0.001
PV V_max_ (cm/s)	91.7 (35.7)	94.5 (32.7)	92.7 (34.7)	0.0849
RA area (cm^2^)	18.2 (5.25)	19.6 (5.88)	18.6 (5.51)	< 0.001
RAP (mmHg)	5.94 (3.33)	6.67 (4.04)	6.18 (3.60)	< 0.001
RV systolic dysfunction, n (%)	373 (16.9%)	149 (13.1%)	522 (15.6%)	0.0159
RVSP (mmHg)	35.8 (10.1)	39.7 (13.1)	37.1 (11.4)	< 0.001
S prime (cm/s)	11.9 (2.63)	10.9 (2.20)	11.6 (2.54)	< 0.001
SV (mL)	84.8 (22.6)	70.1 (26.9)	79.8 (25.1)	< 0.001
SVi (mL/m^2^)	44.2 (11.4)	37.3 (14.1)	41.8 (12.8)	< 0.001
**Comorbidities**				
AF or atrial flutter, n (%)	545 (24.6%)	457 (40.1%)	1002 (29.9%)	< 0.001
Arrhythmias, n (%)	237 (10.7%)	274 (24.0%)	511 (15.2%)	< 0.001
Atherosclerosis, CAD and PVD, n (%)	591 (26.7%)	429 (37.6%)	1020 (30.4%)	< 0.001
Bicuspid, n (%)	192 (8.7%)	29 (2.5%)	221 (6.6%)	< 0.001
Calcified, n (%)	1412 (63.8%)	423 (37.1%)	1835 (54.7%)	< 0.001
Cancer, n (%)	365 (16.5%)	203 (17.8%)	568 (16.9%)	0.641
Cardiomyopathy, n (%)	682 (30.8%)	108 (9.5%)	790 (23.6%)	< 0.001
CKD, n (%)	464 (21.0%)	365 (32.0%)	829 (24.7%)	< 0.001
Conduction disorder, n (%)	212 (9.6%)	129 (11.3%)	341 (10.2%)	0.295
Congenital heart disease, n (%)	22 (1.0%)	12 (1.1%)	34 (1.0%)	0.988
CVA, n (%)	159 (7.2%)	189 (16.6%)	348 (10.4%)	< 0.001
Diabetes with complications, n (%)	710 (32.1%)	526 (46.1%)	1236 (36.9%)	< 0.001
Diabetes without complications, n (%)	309 (14.0%)	331 (29.0%)	640 (19.1%)	< 0.001
Dialysis, n (%)	243 (11.0%)	244 (21.4%)	487 (14.5%)	< 0.001
Heart block, n (%)	33 (1.5%)	99 (8.7%)	132 (3.9%)	< 0.001
Heart failure, n (%)	463 (20.9%)	604 (52.9%)	1067 (31.8%)	< 0.001
Hyperlipidemia, n (%)	327 (14.8%)	181 (15.9%)	508 (15.2%)	0.711
Hypertension, n (%)	1409 (63.7%)	815 (71.4%)	2224 (66.3%)	< 0.001
Hypotension, n (%)	390 (17.6%)	370 (32.4%)	760 (22.7%)	< 0.001
IHD, n (%)	394 (17.8%)	251 (22.0%)	645 (19.2%)	0.0143
Acute coronary syndrome, n (%)	577 (26.1%)	490 (42.9%)	1067 (31.8%)	< 0.001
Nonrheumatic aortic valve disease, n (%)	259 (11.7%)	143 (12.5%)	402 (12.0%)	0.785
Nonrheumatic mitral valve disease, n (%)	35 (1.6%)	56 (4.9%)	91 (2.7%)	< 0.001
Obesity, n (%)	843 (38.1%)	437 (38.3%)	1280 (38.2%)	0.994
Pacemaker, n (%)	120 (5.4%)	101 (8.9%)	221 (6.6%)	< 0.001
PE, n (%)	101 (4.6%)	107 (9.4%)	208 (6.2%)	< 0.001
Pulmonary hypertension, n (%)	131 (5.9%)	144 (12.6%)	275 (8.2%)	< 0.001
Rheumatic heart disease, n (%)	87 (3.9%)	51 (4.5%)	138 (4.1%)	0.76
Sinus bradycardia, n (%)	99 (4.5%)	126 (11.0%)	225 (6.7%)	< 0.001
Sinus tachycardia, n (%)	53 (2.4%)	200 (17.5%)	253 (7.5%)	< 0.001
Syncope, n (%)	165 (7.5%)	224 (19.6%)	389 (11.6%)	< 0.001

Continuous variables reported as mean (standard deviation). Categorical variables reported as frequency (proportion). AF, atrial fibrillation; AR, aortic regurgitation; AV, aortic valve; AVA, aortic valve area; AVAi, indexed aortic valve area; BMI, body mass index; BP, blood pressure; CAD, coronary artery disease; CKD, chronic kidney disease; CVA, cerebrovascular accident; DSI, dimensionless severity index; IHD, ischemic heart disease; LA, left atrium; LVEDD, left ventricular end diastolic diameter; LVEDV, left ventricle end-diastolic volume; LVEF, left ventricular ejection fraction; LVOT, left ventricular outflow tract; LV PW, end-diastolic left ventricular posterior wall thickness; LV SEPT DIAS, LV septal end diastole; MI, myocardial infarction; MPG, aortic valve mean pressure gradient; MR, mitral regurgitation; MV, mitral valve; MS, mitral stenosis; NSTEMI, Non-ST elevation myocardial infarction; PE, pulmonary embolism; PVD, peripheral vascular disease; RAP, right atrial pressure; RV, right ventricle; RVSP, right ventricular systolic pressure; STEMI, ST elevation myocardial infarction; SV, stroke volume; SVi, stroke volume index; TR, tricuspid regurgitation; PV, pulmonary valve; RA, right atrium; UA, unstable angina; V_max_, maximum velocity; VTI, velocity time integral

## Results

### Characteristics of the derivation and external validation cohorts

**Fig. 1:**
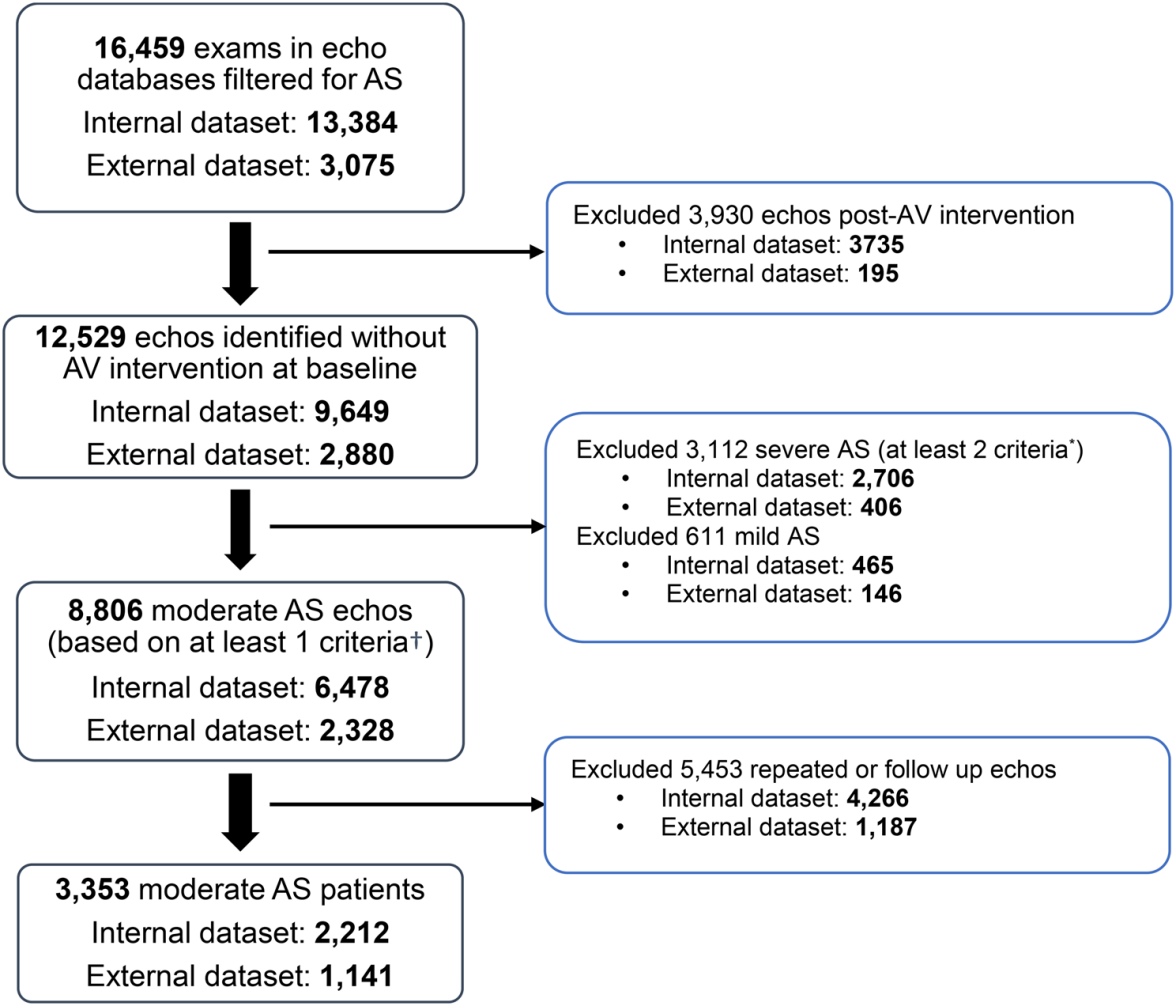
Flow diagram for patient inclusion. AS, aortic stenosis; AV, aortic valve. ^*^Severe AS was based on meeting at least 2 of the following criteria: 1 of aortic valvular area (AVA) < 1 cm^2^ or indexed AVA < 0.6 cm^2^/m^2^, peak velocity ≥4 m/s, AV mean pressure gradient (MPG) ≥40 mmHg, or dimensionless severity index (DSI) < 0.25. ^†^Moderate AS was based on meeting at least 1 of AVA 1.00-1.49 cm^2^, indexed AVA 0.60–0.85 cm^2^/m^2^, peak velocity 3.00–3.99 m/s, MPG 25–39 mmHg or DSI 0.25–0.49

Out of 3,331 patients identified with moderate AS (mean [standard deviation (SD)]: age 73.4 [11.0] years, 65.7% male, MPG 19.1 [7.4] mmHg, AVA 1.4 [0.3] cm^2^), we selected a total of 2,212 patients who had moderate AS on their first echo – those who were already under follow-up were excluded. This group had a median follow-up of 4.3 years (interquartile range: 1.7, 5), and formed the derivation cohort to develop the risk score (Fig. 1). Furthermore, 1,141 patients were included in the external validation cohort.

The baseline characteristics of the derivation and external validation cohorts are summarized in Table 1 (characteristics prior to imputation were summarized in Supplementary Table 4). There were significant differences in most baseline demographic, echocardiographic and clinical characteristics between the two cohorts, such as the derivation cohort having more males, higher MPG, higher peak velocity, higher blood pressures, higher LVEF, but lower proportions of cardiovascular comorbidities such as heart failure at baseline, ischemic heart disease, myocardial infarction, which demonstrates distinctiveness of populations between institutions. Patients in the derivation cohort had lower risk of composite of HF hospitalization or cardiac death at 5 years (27.8% vs. 50.1% in validation cohort, *p* < 0.001), when censored at severe AS or AV intervention **(**Table 2**)**. 4.7% of patients were censored at severe AS or AV intervention. This lower risk of outcomes was consistent without censorship (31.5% vs. 56.9%, *p* < 0.001). Baseline characteristics of the internal training sets of the derivation cohort [internal training set (*n* = 1,772) and internal test dataset (*n* = 442)], were similar and described in Supplementary Table 5.

**Table 2:** Number of events during the follow-up among patients with moderate aortic stenosis

Outcomes (censored at AV intervention and severe AS)	Derivation cohort	External validation cohort	Overall	*P*-value
	(*N* = 2212)	(*N* = 1141)	(*N* = 3353)	
Follow up duration in years, mean (SD)	3.37 (1.82)	1.99 (1.94)	2.90 (1.97)	< 0.001
**Primary outcome**				
5-yr composite of cardiac death or HF hospitalization	614 (27.8%)	572 (50.1%)	1186 (35.4%)	< 0.001
**Secondary outcomes**				
5-year all-cause death	592 (26.8%)	469 (41.1%)	1061 (31.6%)	< 0.001
5-year cardiac death	242 (10.9%)	215 (18.8%)	457 (13.6%)	< 0.001
5-year HF hospitalization	763 (34.5%)	542 (47.5%)	1305 (38.9%)	< 0.001
5-year progression to severe AS	397 (17.9%)	227 (19.9%)	624 (18.6%)	0.39
**5-year HF hospitalization (death as competing risk)**				
no event	1217 (55.0%)	409 (35.8%)	1626 (48.5%)	< 0.001
heart failure	763 (34.5%)	542 (47.5%)	1305 (38.9%)	
death	232 (10.5%)	190 (16.7%)	422 (12.6%)	
**5-year cardiac death (death as competing risk)**				
no event	1620 (73.2%)	672 (58.9%)	2292 (68.4%)	< 0.001
cardiac death	242 (10.9%)	215 (18.8%)	457 (13.6%)	
death from other causes	350 (15.8%)	254 (22.3%)	604 (18.0%)	
**5-year progression to severe AS (AV intervention and death as competing risk)**				
no event	1160 (52.4%)	425 (37.2%)	1585 (47.3%)	< 0.001
severe AS	393 (17.8%)	217 (19.0%)	610 (18.2%)	
AV intervention or death	659 (29.8%)	499 (43.7%)	1158 (34.5%)	
**Interventions**				
5-yr AV intervention	187 (8.5%)	93 (8.2%)	280 (8.4%)	0.956
1-yr AV Intervention	52 (2.4%)	46 (4.0%)	98 (2.9%)	0.0236

AV, aortic valve; AS, aortic stenosis; SD, standard deviation

### Derivation of the predictive model

For simplicity and to prevent overfitting after performing LASSO and greedy algorithm with 17 variables, the 10 most significant variables based on lowest *p* values followed by highest beta coefficients were selected: age, left ventricular end-diastolic diameter (LVEDD), LVOT VTI, mitral valve E-wave, end-diastolic left ventricular posterior wall (LV PW) thickness, moderate/severe mitral regurgitation, moderate/severe tricuspid regurgitation, diastolic blood pressure, acute coronary syndrome, and hyperlipidemia. A sensitivity analysis (see below) was performed showing negligible impact of excluding the lowest ranked 7 variables (AVA, pulmonary valve peak velocity, LVEF, non-rheumatic AV disease, calcification, dialysis, and diabetes with complications). Table 3 summarizes the HR for each of the top 10 variables to predict risk score assignment in a multivariable Cox proportional hazard model. The proportional hazard assumption in the Cox model was tested using plots over time (Supplementary Fig. 1).

**Table 3:** Hazard ratios from multivariable regression model for 5-year composite outcome

Characteristic	Adjusted HR	95% CI	*p*
Age (years)	1.02	1.01, 1.03	< 0.001
Diastolic blood pressure (mmHg)	0.98	0.97, 0.99	< 0.001
E-wave (cm/s)	1.01	1.01, 1.01	< 0.001
End-diastolic left ventricular posterior wall thickness (cm)	2.92	1.84, 4.63	< 0.001
Left ventricular end diastolic diameter (cm)	1.35	1.18, 1.53	< 0.001
Left ventricular outflow tract velocity time integral (cm)	0.97	0.95, 0.99	< 0.001
Moderate or severe mitral regurgitation	1.55	1.17, 2.06	0.002
Moderate or severe tricuspid regurgitation	1.45	1.06, 1.98	0.019
Hyperlipidemia	1.46	1.17, 1.83	< 0.001
Acute coronary syndrome	1.46	1.20, 1.77	< 0.001

CI, confidence interval; HR, hazard ratio

### Performance of the model

The *C*-statistic for composite outcome within 5-years was 0.70 (95% CI: 0.67–0.76) in the internal validation dataset and 0.75 (95% CI: 0.70–0.79) in the external validation set, demonstrating good predictive performance (Supplementary Fig. 2A). The time-dependent ROC curves for the external validation set also demonstrated *C*-statistics of 0.76–0.78 (Supplementary Fig. 2B).

### Association of risk score and composite outcome

**Fig. 2:**
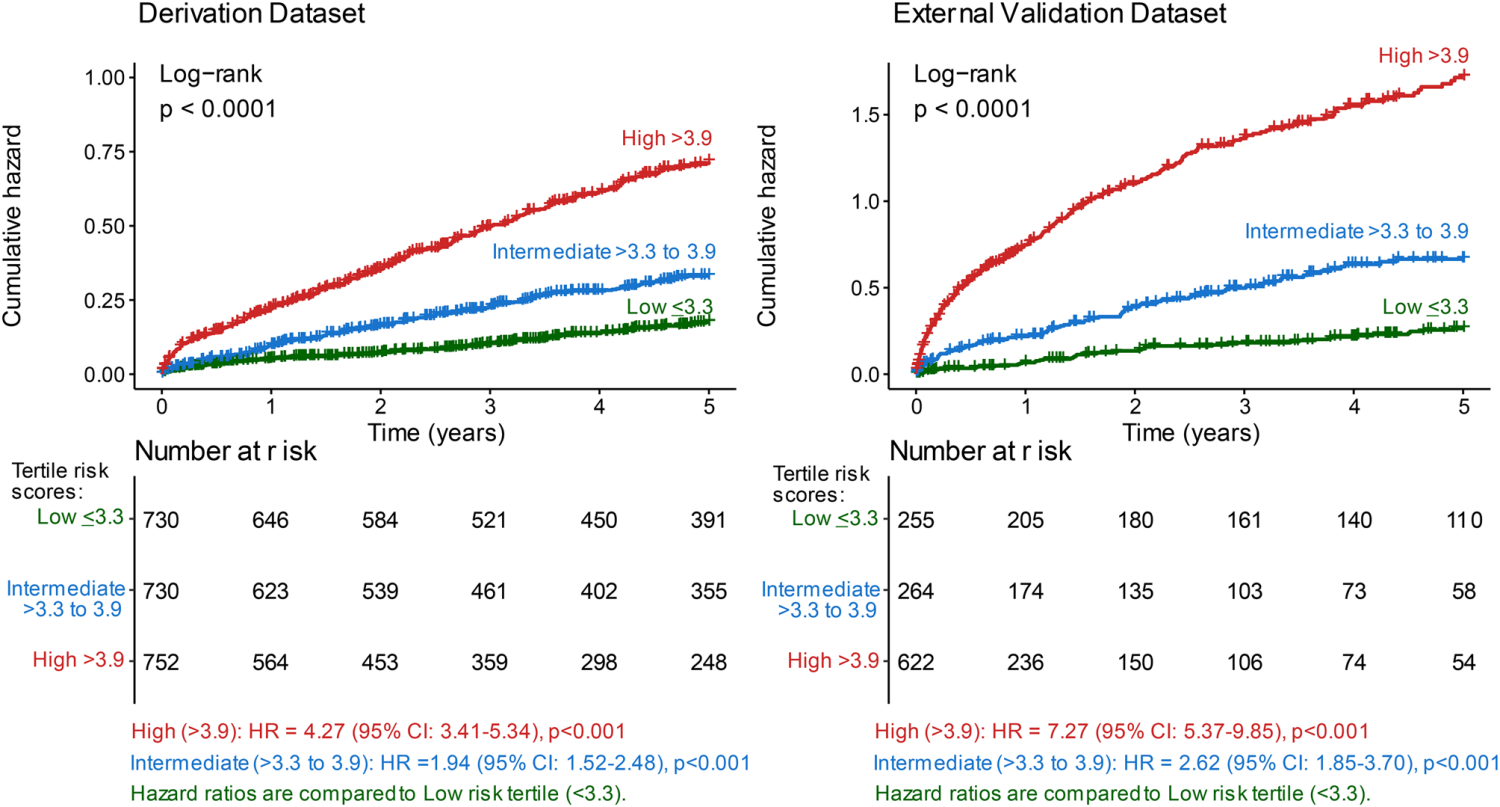
Cumulative hazard plots comparing composite outcome in patients with moderate aortic stenosis

The relationships between score tertiles with composite outcome are illustrated in Fig. 2 using cumulative hazard plots and survival rates (Supplementary Table 6A) with good separation of curves and survival rates between all tertiles. The proportion of composite outcome was highest in the highest risk-score group (score > 3.9) and lowest in the lowest risk-score group (score < 3.3). Similarly, the HR increased with risk score and was highest in tertile 3 in both the derivation and external validation cohorts. A high risk-score (> 3.9) was associated with over 4 times higher risk in both the derivation (HR: 4.27, 95% CI: 3.41–5.34, *p* < 0.001) and external validation cohorts (HR: 7.27, 95% CI: 5.37–9.85, *p* < 0.001) compared with a low risk-score (< 3.3) (Supplementary Table 6B).

The baseline characteristics and outcomes were compared between the risk score groups (Supplementary Table 7). Patients with a high risk-score (> 3.9) in both the derivation and external validation datasets were older (mean 78–79 years), had lower MPG or peak velocity, lower indexed stroke volume, lower LVEF, more with RV systolic dysfunction and/or dilated RV, and higher proportions of CV comorbidities, especially heart failure at baseline, hypertension, atrial fibrillation and other valvular heart diseases. More patients in the low risk-score (< 2) group had AV intervention within 5 years. The proportions of composite, secondary outcomes (all-cause death, cardiac death, heart failure hospitalization), cardiac death (with all-cause death as competing risk), and HF hospitalization (with all-cause death as competing risk) were all consistently highest in the high risk-score (> 3.9) group, followed by intermediate risk-score (3.3–3.9) group, and lowest in the low risk-score (< 3.3) group. Hazard ratios were also determined for the secondary outcomes and competing risk analyses (Supplementary Table 6 C). The risks of all-cause death, cardiac death, and heart failure hospitalization were consistently highest in the high risk-score (> 3.9) group, followed by intermediate risk-score (3.3–3.9) group.

### Sensitivity analysis

The LASSO method selected 22 variables to be included in the model, while after the greedy selection algorithm, a total of 17 variables were included (Supplementary Table 8). *C*-statistic for composite outcome within 5 years did not improve with additional variables (*C*-statistic 0.76 (95% CI: 0.71–0.81) in the external validation set) (Supplementary Fig. 3A). Similarly, the time-dependent *C*-statistic from within 1 to 5 years ranged from 0.77 to 0.80 (Supplementary Fig. 3B).

### Use of model and web application

**Fig. 3:**
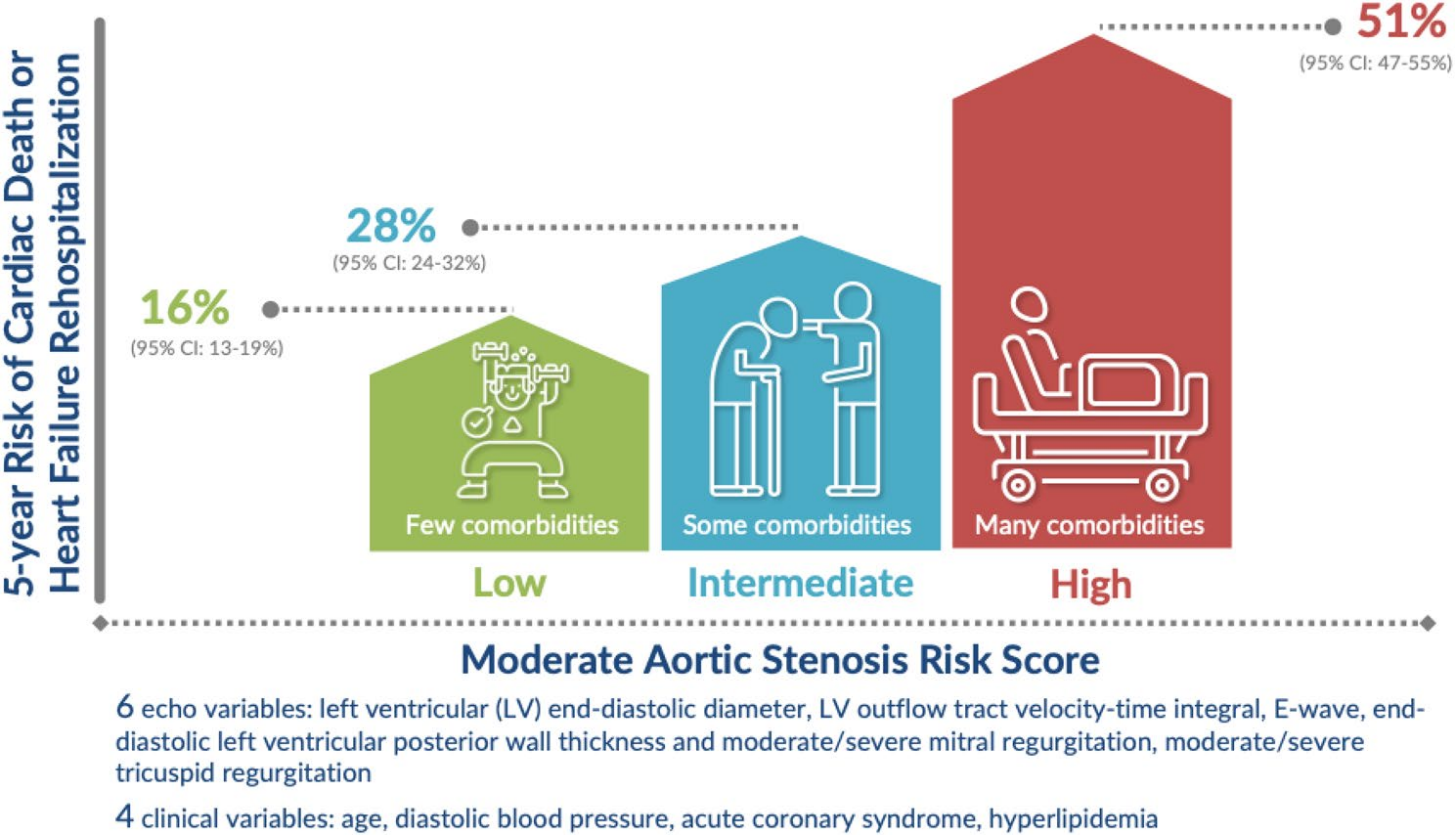
Demonstrative adverse outcomes in patients with moderate aortic stenosis managed with conservative therapy. Each of three risk scenarios (low, intermediate, and high risk) depicts 10 patients, illustrating varying degrees of comorbidities and associated risk

Based on the final model, a Moderate Aortic Stenosis Risk Calculator was developed: https://baker-biostats.shinyapps.io/AS_risk_calculator/. This calculator leverages the final Cox model generated within this study to provide a clinician with an estimated composite risk from conservative management. Adverse outcomes in patients with moderate AS who were treated conservatively is shown in Fig. 3 across low-, intermediate-, and high-risk scenarios.

## Discussion

This study derived and validated a risk score to stratify prognosis in patients with moderate AS. This risk score incorporates a total of 10 components (Central illustration), based on 6 echocardiographic variables (LVEDD, LVOT VTI, LV PW, mitral valve E-wave, moderate/severe mitral regurgitation, and moderate/severe tricuspid regurgitation) and 4 clinical variables (age, acute coronary syndrome, diastolic blood pressure, and hyperlipidemia).

### Variables associated with outcome in moderate AS

The following echocardiographic parameters individually or in combination have been shown in our study and in others to be associated with adverse outcomes in AS. LVEDD is a measure of the size of the LV at the end of diastole, reflecting the extent of ventricular dilation [28]. This dilation often signifies increased volume overload and compromised systolic function, leading to a higher risk of adverse events such as cardiac death. A larger LVEDD therefore indicates more significant left ventricular dilation, which is associated with poorer health outcomes [28]. Although LVEDD enlargement is not a classic hallmark of isolated aortic stenosis, when present it usually signals concomitant cardiac pathology, such as post‑myocardial‑infarction remodeling, secondary mitral regurgitation, or chronic pressure‑volume mismatch, that further worsens prognosis. LV PW and LVEDD are used to calculate LV mass index (LVMI). A higher LVMI is associated with LV hypertrophy, which is commonly seen in AS, and is also linked to increased risk of adverse outcomes [29]. LVMI and relative wall thickness were less predictive than LV PW and LVEDD in our model assessment. These latter variables provide a more specific measure of LV hypertrophy and its impact on AS prognosis. LVOT VTI is a measure of cardiac output and stroke volume. A reduced LVOT VTI reflects lower stroke volume, which can be indicative of reduced left ventricular function [30]. This parameter is directly related to prognosis, as a lower LVOT VTI suggests impaired cardiac performance and is associated with increased mortality [31]. The mitral E-wave is used in assessment of LV diastolic function and reflects the pressure gradient between left atrium and LV during early diastole and affected by alterations in rate of LV relaxation [32]. As the severity of AS worsens, the LV becomes stiffer, leading to impaired diastolic filling and elevated left atrial pressure (LAP) [33]. This increased LAP is reflected by a higher E wave velocity, which suggests worsening diastolic dysfunction and elevated filling pressures, which can be associated with poorer clinical outcomes. In our study, a higher E wave was associated with adverse outcomes in AS, although the hazard ratio approached 1 for each increment of 1 cm/s. The presence of moderate or severe mitral or tricuspid regurgitation are also indicative of multi-valvular disease, which compounds the severity of AS, exacerbates heart failure symptoms and increases risk of death [34].

In patients with AS, comorbidities such as lower diastolic blood pressure can be linked with poorer outcomes [35]. In addition to lower overall blood pressure signalling compromised cardiac function, low coronary perfusion pressure may compromise myocardial perfusion, which occurs during diastole, and both may contribute to the increased risk of adverse outcomes [35]. Acute coronary syndrome impacts the heart’s ability to pump effectively and can exacerbate the progression of AS [36]. The presence of acute coronary syndrome is a significant risk factor for adverse outcomes, as it contributes to further myocardial damage and compromised cardiac function. Elevated lipid levels are a well-established risk factor for acute coronary syndrome, which in turn accelerates the progression of AS [37]. Hyperlipidemia also leads to atherosclerosis and coronary artery disease, which can further impair cardiac function and worsen patient outcomes [37]. Advanced age is a robust predictor of both cardiac death and HF in patients with AS [38]. Older age is associated with reduced physiological reserve and increased comorbidities, which collectively contribute to a higher risk of adverse outcomes.

Overall, the risk of moderate AS seems to be between that of mild and severe AS [39], despite some studies demonstrating similar outcomes in moderate and severe AS [2]. Some of this heterogeneity may arise from measurement inaccuracy, whereby patients with truly severe AS are potentially misidentified as having moderate AS instead. Among patients with true moderate AS, variability in outcomes appears to be driven by concurrent non-valvular cardiac disease (left ventricular dysfunction and coronary disease) [40], and non-cardiac multi-morbidity [41]. Adverse events over follow-up may be more reflective of comorbidities rather than AV disease. Despite this variability in outcomes, early intervention in moderate AS has been associated with a reduction of adverse events in observational studies [42, 43]. Prospective trials in moderate AS are being undertaken to clarify this association [44].

### Variable selection

The Moderate Aortic Stenosis Risk Calculator was developed as a comprehensive clinical tool for integrating valvular, cardiac and non-cardiac parameters and to generate a patient risk profile. Understanding and quantifying risk profiles is important for patient care (e.g. shared decision-making about interventions and frequency of follow-up) and to ensure balanced risk profiles between randomized groups in clinical trials. The results in the validation group confirmed that our combination of parameters accurately identified the risk of cardiovascular death and HF hospitalization in moderate AS, with an area under the receiver operating characteristic curve of 0.75 (95% CI: 0.70–0.79). The risk score divided into tertiles demonstrated differential outcomes in cardiac death, all-cause death, and heart failure hospitalization.

In recent years, machine learning techniques have emerged as promising tools for developing predictive models for cardiovascular care, by integrating highly dimensional data [45]. Among these techniques, LASSO, a supervised machine learning algorithm, offers an attractive solution. This method performs feature selection and simultaneously shrinks the coefficients that do not contribute significantly to predicting the outcome, thereby reducing risk of overfitting and improving model interpretability [46]. However, the derivation of risk assessment tools from big data requires validation and quality control – the use of heterogenous data sources that lack standardization in parameter and outcome definitions can undermine the generalizability of risk prediction [9]. By leveraging machine learning and quality-controlled data, this study aimed to develop and validate a risk score by integrating demographic, clinical, and imaging data for predicting adverse outcomes in patients with moderate AS.

Despite the benefits of a decision support tool, clinical decisions regarding AV intervention remain complex and cannot be solely based on a single factor, such as a risk score. We identified the ten strongest variables of the composite endpoint, but some other relevant variables such as sex and atrial fibrillation were removed during the penalization step in the machine learning approach. Although both variables were included in the initial candidate set, their coefficients were reduced toward zero, suggesting that, after adjusting for more dominant factors, they contributed little additional prognostic information in this cohort. This does not rule out clinically meaningful sex‑specific effects or an impact of AF on other outcomes (e.g., stroke). A detailed and thorough discussion between the clinician and the patient that considers a variety of factors such as patient preferences, overall health status, and the potential risks and benefits of intervention should be regarded as best practice. Our risk score is intended to inform these discussions rather than to dictate a specific course of action or management.

### Previous risk assessment approaches

The results of this study build on previous reports of risk scores that have identified patterns of comorbidities among patients with AS (Supplementary Table 9) [47–54]. Previous studies have focused on developing risk prediction models for patients with AS, particularly with respect to mortality and adverse outcomes following AV intervention. Three studies focused on predicting outcomes after AV intervention for severe AS [48, 52]. Two were single center design, thus limiting generalizability, and none had an external validation cohort. Three additional single center studies included mostly severe AS also without external validation [51]. Only two previous studies address risk prediction in moderate AS. Holme et al. [53] developed a seven-factor model for risk stratification of asymptomatic mild-to-moderate AS patients; however, this study was limited to patients enrolled in the Simvastatin and Ezetimibe in Aortic Stenosis trial, without external validation. Namasivayam et al. [49] developed the Aortic Stenosis Risk (ASteRisk) score to predict adverse outcomes for patients with at least moderate AS. Generalizability to patients undergoing conservative management is limited given patients who underwent AV intervention during the follow-up period of 5 years were included. As the final 9 variables (namely, energy loss, AVA, transvalvular flow rate, MPG, hyperlipidemia, myocardial infarction, peripheral vascular disease, wall motion abnormality, posterior wall thickness, chronic kidney disease, chronic HF) out of 26 selected from bootstrap LASSO regression for inclusion in their model were hand-selected by the authors the impact of selection bias is uncertain. Their model was also based on a smaller cohort evaluated over a decade ago, and its value in the current era of AS patients is unclear.

### Strengths and limitations

Our study has several strengths. We have addressed the limitations of previous studies - including the need for external validation, potential biases inherent in study design with the sample size, inclusion and exclusion criteria, and the validation of echocardiographic parameters. Notably, our predictive risk score was derived from a sample size of over 2,000 patients with a follow-up period of five years, which enhanced the statistical power and precision of the analyses. Leveraging multiple linked data sources effectively mitigated the risk of missing data. Furthermore, the blinded manual validation of individual echocardiographic parameters ensures the reliability and validity of the measurements utilized in the external cohort. Evaluation of the risk score with an external validation dataset from a distinct geographic region enhanced the generalizability of the findings. The study populations were culturally diverse and incorporated a wide variety of socioeconomic status, thereby increasing confidence in the applicability of the risk score beyond the derivation cohort. While there are differences between the derivation and external validation cohorts, the predictive value of the risk score in the external validation dataset supports broad application in diverse populations.

Limitations of our study are generic to “Big Data” cohorts, including the fact that it pertains to practice in previous years and a particular jurisdiction – it may not be generalizable to non-Australian patient cohorts. Other limitations include potential unmeasured confounders, bias associated with clinical decision-making, and misclassification bias related to reliance on ICD-10 codes. Echocardiographic parameters in the internal cohort were not independently validated. From a cardiac perspective, our risk score helps quantify the longitudinal risks associated with moderate AS – we censored patients when they developed severe AS, as the management of this entity is generally accepted. In this respect, it may be useful in guiding clinical decision-making as indications for AV intervention continue to expand. However, it is important to highlight that randomized controlled trials, which are ongoing at this time, are needed to definitively determine if early AV intervention is beneficial for patients with high-risk scores. The study also lacked a central adjudication committee, so variability in data interpretation across participating sites could not be uniformly controlled. Furthermore, although LV strain and left atrial strain are associated with outcomes, these were not captured in these datasets.

## Conclusion

The results of this study indicate that risk can be quantified in moderate, non-intervened AS. Clinical, demographic and echocardiographic factors were found to represent different aspects of cardiac function and pathology, as well as influence patient outcomes in this cohort. Understanding the level of risk will facilitate decision-making in this heterogeneous group of patients and help to better predict outcomes and guide clinical management.

## Supplementary Information

Below is the link to the electronic supplementary material.

Supplementary Material 1

## Data Availability

The data that support the findings of this study are available from Queensland Cardiac Outcomes Registry and the Centre for Victorian Data Linkage Unit (CVDL), but restrictions apply to the availability of these data, which were used within data linkage program under ethics for the current study, and so are not publicly available.

## Abbreviations

AS: Aortic stenosis
AVA: Aortic valve area
AV: Aortic valve
CI: Confidence interval
CVDL: Centre for Victorian Data Linkage
DSI: Dimensionless severity index
HR: Hazard ratio
ICD-10: International Classification of Diseases 10th Edition
LA: Left atrium
LASSO: Least absolute shrinkage and selection operator
LVEF: Left ventricular ejection fraction
LVOT: Left ventricular outflow tract
LV PW: End-diastolic left ventricular posterior wall thickness
MPG: Mean pressure gradient
QCOR: Queensland Cardiac Outcomes Registry
SD: Standard deviation
VTI: Velocity time integral
