# Supplementary material for: Clinical risk score for cardiac death or heart failure hospitalization in moderate aortic stenosis
Source: Echo Res Pract. 2026 Mar 16;13:8. doi: 10.1186/s44156-026-00110-w (PMC12990625; doi:10.1186/s44156-026-00110-w)
Supplement: Supplementary file 1 — Supplementary Material 1 [file 44156_2026_110_MOESM1_ESM.docx]

**Supplementary Materials**

| **Supplementary Table 1. Outcome definitions** |
| --- |
| **Cardiac death** was extracted from death registries in Queensland Cardiac Outcomes Registry (QCOR) or Centre for Victorian Data Linkage (CVDL) by text-mining causes of death including heart disease, myocardial infarct, coronary artery disease, acute coronary syndrome, cardiopulmonary failure, ischaemic heart disease, sudden cardiac death, cardiogenic shock, heart failure, cardiomyopathy, cardiac amyloidosis, coronary atherosclerosis, ventricular tachycardia, ventricular fibrillation, endocarditis, septicemia from aortic valve (AV), poor or reduced left ventricular ejection fraction (LVEF), cardioembolism from mitral valve replacement, heart block, valvular heart disease, cardiac valvular disease, heart attack, poor cardiac function, ventricular failure, coronary obstruction, myopericarditis, pericardial effusion, cardio-renal syndrome, aortic regurgitation, AS, valve stenosis, bicuspid AV, mitral stenosis, AV disease, AV and pulmonary valve replacements, aortic and mitral valve replacement, or recent AV surgery in high risk. |
| **Heart failure** was defined as one or more hospitalizations from admitted episode datasets from QCOR or CVDL extracted from International Classification of Diseases 10^th^ Edition (ICD-10) codes: I110, I130, I132, I50, I500, I501, I509, U822, or I255 or had left ventricular failure, heart failure, ischemic cardiomyopathy recorded during admission, or if they had severe AS, heart failure was also defined as one or more hospitalizations with ICD-10 codes: J81, J9600, J9601, J9609, R060, or had pulmonary oedema, dyspnea, acute respiratory failure documented during admission. |

**Supplementary Table 2. List of predictor variables assessed:**

- Acute coronary syndrome includes ST elevation myocardial infarction, Non-ST elevation myocardial infarction, unstable angina
- Age
- Aortic valve area
- Aortic valve mean gradient
- Aortic valve peak velocity
- Aortic valve velocity time integral
- Arrhythmias include ventricular fibrillation, ventricular tachycardia, cardiac arrest, or supraventricular tachycardia
- Atherosclerosis, coronary artery disease and peripheral vascular disease
- Atrial fibrillation or atrial flutter
- Bicuspid
- Body mass index
- Calcified
- Cancer
- Cardiomyopathy
- Cerebral vascular accident
- Chronic kidney disease
- Conduction disorder
- Congenital heart disease
- Diabetes with complications
- Diabetes without complications
- Dialysis
- Diastolic blood pressure
- Dilated right ventricle
- Dimensionless severity index
- E/A
- E/e'
- End-diastolic left ventricular posterior wall thickness
- heart block
- Heart failure
- Height
- Hyperlipidaemia
- Hypertension
- Hypotension
- Ischemic heart disease includes stable angina
- Left atrium volume
- Left ventricular ejection fraction
- Left ventricular end diastolic diameter
- Left ventricular outflow tract diameter
- Left ventricular outflow tract velocity time integral
- Left ventricular septal end diastole
- Male
- Mitral valve deceleration time
- Mitral valve E wave
- Moderate or Severe aortic regurgitation
- Moderate or Severe mitral regurgitation
- Moderate or severe mitral regurgitation
- Moderate or Severe mitral stenosis
- Moderate or Severe tricuspid regurgitation
- Moderate or severe tricuspid regurgitation
- Nonrheumatic aortic valve disease
- Nonrheumatic mitral valve disease
- Obesity
- Pacemaker
- Pulmonary embolism
- Pulmonary hypertension
- Pulmonary valve peak velocity
- Rheumatic heart disease
- Right atrial pressure
- Right atrium area
- Right ventricle systolic dysfunction
- Right ventricular systolic pressure
- S prime
- Sinus bradycardia
- Sinus tachycardia
- Syncope
- Systolic blood pressure
- Weight

**Supplementary Table 3. Variables excluded due to >20% missing data:**

| **Variable** | **Missing (%)** |
| --- | --- |
| RVSP | 39.2 |
| E/e’ | 23.7 |
| E/A | 35.8 |
| TAPSE | 50.9 |
| S’ | 27.9 |
| RAP | 34.4 |

**Supplementary Table 4.** Baseline characteristics of patients with moderate aortic stenosis in derivation and external validation cohorts prior to imputation.

|  |  | **Derivation cohort (N=2212)** | **External validation (N=1141)** | **Overall (N=3353)** | **P-value** |
| --- | --- | --- | --- | --- | --- |
| **Age (years)** | Mean (SD) | 73.4 (11.0) | 73.9 (13.5) | 73.6 (11.9) | 0.517 |
|  | Median [Q1, Q3] | 74.6 [67.1, 81.3] | 77.0 [68.0, 83.0] | 75.3 [67.2, 82.0] |  |
|  | [Min, Max] | [20.2, 103] | [18.0, 98.0] | [18.0, 103] |  |
| **Male** | 0 | 758 (34.3%) | 556 (48.7%) | 1314 (39.2%) | <0.001 |
|  | 1 | 1454 (65.7%) | 585 (51.3%) | 2039 (60.8%) |  |
| **Height (cm)** | Mean (SD) | 169 (9.90) | 163 (11.9) | 167 (10.9) | <0.001 |
|  | Median [Q1, Q3] | 170 [162, 176] | 165 [156, 170] | 167 [160, 175] |  |
|  | [Min, Max] | [100, 200] | [58.0, 201] | [58.0, 201] |  |
| **Weight (kg)** | Mean (SD) | 84.0 (19.8) | 80.2 (20.6) | 82.7 (20.1) | <0.001 |
|  | Median [Q1, Q3] | 82.0 [70.0, 95.0] | 79.0 [67.0, 90.0] | 81.0 [70.0, 93.0] |  |
|  | [Min, Max] | [34.0, 190] | [34.0, 235] | [34.0, 235] |  |
| **BMI (kg/m^2^)** | Mean (SD) | 28.1 (9.80) | 28.9 (20.6) | 28.4 (14.4) | 0.374 |
|  | Median [Q1, Q3] | 28.2 [24.4, 32.4] | 28.1 [24.3, 33.1] | 28.2 [24.4, 32.7] |  |
|  | [Min, Max] | [0.00158, 190] | [0.00215, 365] | [0.00158, 365] |  |
| **Obesity** | 0 | 1369 (61.9%) | 704 (61.7%) | 2073 (61.8%) | 0.994 |
|  | 1 | 843 (38.1%) | 437 (38.3%) | 1280 (38.2%) |  |
| **RVSP (mmHg)** | Mean (SD) | 37.3 (12.6) | 40.8 (14.6) | 38.8 (13.5) | <0.001 |
|  | Median [Q1, Q3] | 35.0 [28.0, 44.0] | 38.0 [30.0, 49.9] | 36.0 [29.0, 46.0] |  |
|  | [Min, Max] | [1.00, 117] | [8.00, 102] | [1.00, 117] |  |
|  | Missing | 1000 (45.2%) | 315 (27.6%) | 1315 (39.2%) |  |
| **E/e'** | Mean (SD) | 8.39 (7.67) | 15.3 (5.71) | 11.2 (7.72) | <0.001 |
|  | Median [Q1, Q3] | 8.94 [0.183, 13.9] | 14.3 [11.0, 18.9] | 11.6 [6.51, 16.3] |  |
|  | [Min, Max] | [0.0413, 29.7] | [0.0500, 30.0] | [0.0413, 30.0] |  |
|  | Missing | 767 (34.7%) | 169 (14.8%) | 936 (27.9%) |  |
| **E/A** | Mean (SD) | 1.02 (0.545) | 1.09 (0.598) | 1.05 (0.566) | 0.0165 |
|  | Median [Q1, Q3] | 0.865 [0.700, 1.14] | 0.900 [0.715, 1.24] | 0.890 [0.700, 1.20] |  |
|  | [Min, Max] | [0.300, 4.33] | [0.0200, 4.41] | [0.0200, 4.41] |  |
|  | Missing | 864 (39.1%) | 344 (30.1%) | 1208 (36.0%) |  |
| **TAPSE (cm)** | Mean (SD) | 2.09 (0.498) | 1.96 (0.503) | 2.02 (0.505) | <0.001 |
|  | Median [Q1, Q3] | 2.10 [1.80, 2.40] | 2.00 [1.60, 2.30] | 2.00 [1.70, 2.40] |  |
|  | [Min, Max] | [0.500, 3.70] | [0.600, 3.50] | [0.500, 3.70] |  |
|  | Missing | 1448 (65.5%) | 273 (23.9%) | 1721 (51.3%) |  |
| **LVDD (cm)** | Mean (SD) | 4.72 (0.727) | 4.87 (0.802) | 4.77 (0.757) | <0.001 |
|  | Median [Q1, Q3] | 4.70 [4.20, 5.20] | 4.81 [4.30, 5.40] | 4.72 [4.24, 5.24] |  |
|  | [Min, Max] | [2.20, 7.60] | [2.30, 7.50] | [2.20, 7.60] |  |
|  | Missing | 123 (5.6%) | 39 (3.4%) | 162 (4.8%) |  |
| **S prime (cm/s)** | Mean (SD) | 12.0 (2.98) | 10.6 (2.78) | 11.6 (2.98) | <0.001 |
|  | Median [Q1, Q3] | 12.0 [10.0, 14.0] | 10.0 [10.0, 11.0] | 11.0 [10.0, 13.0] |  |
|  | [Min, Max] | [3.00, 25.0] | [1.00, 22.5] | [1.00, 25.0] |  |
|  | Missing | 551 (24.9%) | 552 (48.4%) | 1103 (32.9%) |  |
| **RAP (mmHg)** | Mean (SD) | 6.29 (4.08) | 6.83 (4.49) | 6.50 (4.26) | 0.0139 |
|  | Median [Q1, Q3] | 5.00 [3.00, 8.00] | 5.00 [3.00, 8.00] | 5.00 [3.00, 8.00] |  |
|  | [Min, Max] | [1.00, 20.0] | [3.00, 20.0] | [1.00, 20.0] |  |
|  | Missing | 885 (40.0%) | 268 (23.5%) | 1153 (34.4%) |  |
| **Moderate or Severe AR** | 0 | 2138 (96.7%) | 1100 (96.4%) | 3238 (96.6%) | 0.933 |
|  | 1 | 74 (3.3%) | 41 (3.6%) | 115 (3.4%) |  |
| **Moderate or Severe MR** | 0 | 2030 (91.8%) | 974 (85.4%) | 3004 (89.6%) | <0.001 |
|  | 1 | 182 (8.2%) | 167 (14.6%) | 349 (10.4%) |  |
| **Moderate or Severe MS** | 0 | 2196 (99.3%) | 1130 (99.0%) | 3326 (99.2%) | 0.761 |
|  | 1 | 16 (0.7%) | 11 (1.0%) | 27 (0.8%) |  |
| **Moderate or Severe TR** | 0 | 2059 (93.1%) | 978 (85.7%) | 3037 (90.6%) | <0.001 |
|  | 1 | 153 (6.9%) | 163 (14.3%) | 316 (9.4%) |  |
| **RV systolic dysfunction** | 0 | 1839 (83.1%) | 992 (86.9%) | 2831 (84.4%) | 0.0159 |
|  | 1 | 373 (16.9%) | 149 (13.1%) | 522 (15.6%) |  |
| **Dilated RV** | 0 | 2001 (90.5%) | 977 (85.6%) | 2978 (88.8%) | <0.001 |
|  | 1 | 211 (9.5%) | 164 (14.4%) | 375 (11.2%) |  |
| **LVOT diameter (cm)** | Mean (SD) | 2.23 (0.207) | 2.08 (0.216) | 2.18 (0.221) | <0.001 |
|  | Median [Q1, Q3] | 2.20 [2.10, 2.36] | 2.10 [1.95, 2.20] | 2.20 [2.00, 2.30] |  |
|  | [Min, Max] | [1.60, 3.42] | [1.39, 2.82] | [1.39, 3.42] |  |
| **LVOT VTI (cm)** | Mean (SD) | 21.4 (5.25) | 20.5 (7.12) | 21.1 (5.96) | <0.001 |
|  | Median [Q1, Q3] | 21.3 [18.0, 24.2] | 20.2 [15.9, 24.4] | 21.0 [17.4, 24.2] |  |
|  | [Min, Max] | [0.202, 43.8] | [5.12, 103] | [0.202, 103] |  |
| **AV VTI (cm)** | Mean (SD) | 58.8 (16.4) | 48.1 (18.4) | 55.2 (17.8) | <0.001 |
|  | Median [Q1, Q3] | 58.3 [48.0, 68.7] | 45.9 [34.1, 60.9] | 54.9 [43.0, 66.1] |  |
|  | [Min, Max] | [0.700, 133] | [1.58, 109] | [0.700, 133] |  |
| **AV mean gradient (mmHg)** | Mean (SD) | 19.1 (7.43) | 13.4 (8.40) | 17.1 (8.23) | <0.001 |
|  | Median [Q1, Q3] | 18.0 [13.7, 23.0] | 11.4 [6.91, 18.1] | 16.3 [11.0, 22.0] |  |
|  | [Min, Max] | [3.00, 41.0] | [1.24, 39.4] | [1.24, 41.0] |  |
| **AVA (cm^2^)** | Mean (SD) | 1.43 (0.292) | 1.41 (0.317) | 1.42 (0.301) | 0.242 |
|  | Median [Q1, Q3] | 1.40 [1.23, 1.60] | 1.38 [1.27, 1.59] | 1.40 [1.25, 1.60] |  |
|  | [Min, Max] | [0.600, 3.40] | [0, 2.94] | [0, 3.40] |  |
| **AVAi (cm^2^/m^2^)** | Mean (SD) | 0.742 (0.152) | 0.754 (0.182) | 0.746 (0.163) | 0.128 |
|  | Median [Q1, Q3] | 0.732 [0.640, 0.830] | 0.752 [0.649, 0.850] | 0.737 [0.643, 0.838] |  |
|  | [Min, Max] | [0.316, 1.95] | [0, 2.23] | [0, 2.23] |  |
| **AV Vmax (cm/s)** | Mean (SD) | 267 (67.2) | 231 (73.8) | 254 (71.5) | <0.001 |
|  | Median [Q1, Q3] | 266 [220, 312] | 225 [172, 282] | 254 [202, 303] |  |
|  | [Min, Max] | [70.0, 470] | [20.0, 476] | [20.0, 476] |  |
| **DSI** | Mean (SD) | 0.458 (1.17) | 0.464 (0.144) | 0.460 (0.956) | 0.986 |
|  | Median [Q1, Q3] | 0.380 [0.316, 0.437] | 0.442 [0.368, 0.530] | 0.397 [0.328, 0.468] |  |
|  | [Min, Max] | [0.00481, 26.5] | [0.0430, 1.85] | [0.00481, 26.5] |  |
| **Systolic BP (mmHg)** | Mean (SD) | 137 (16.1) | 132 (12.6) | 135 (15.2) | <0.001 |
|  | Median [Q1, Q3] | 137 [128, 146] | 129 [128, 139] | 134 [128, 145] |  |
|  | [Min, Max] | [18.0, 235] | [78.0, 205] | [18.0, 235] |  |
| **Diastolic BP (mmHg)** | Mean (SD) | 73.3 (8.08) | 71.0 (7.28) | 72.5 (7.89) | <0.001 |
|  | Median [Q1, Q3] | 74.5 [68.7, 77.3] | 69.0 [68.4, 75.2] | 73.6 [68.5, 76.8] |  |
|  | [Min, Max] | [33.0, 129] | [8.00, 112] | [8.00, 129] |  |
| **PV Vmax (cm/s)** | Mean (SD) | 91.7 (35.7) | 94.5 (32.7) | 92.7 (34.7) | 0.0849 |
|  | Median [Q1, Q3] | 95.6 [82.5, 110] | 92.8 [90.8, 102] | 93.9 [85.6, 108] |  |
|  | [Min, Max] | [0.300, 240] | [-144, 333] | [-144, 333] |  |
| **MV E wave (cm/s)** | Mean (SD) | 89.6 (34.0) | 97.2 (29.4) | 92.2 (32.7) | <0.001 |
|  | Median [Q1, Q3] | 82.9 [66.9, 107] | 95.6 [75.6, 110] | 86.4 [70.0, 109] |  |
|  | [Min, Max] | [27.6, 300] | [29.8, 263] | [27.6, 300] |  |
| **MV deceleration time (msec)** | Mean (SD) | 239 (67.2) | 220 (63.7) | 232 (66.6) | <0.001 |
|  | Median [Q1, Q3] | 231 [194, 269] | 216 [184, 245] | 222 [190, 261] |  |
|  | [Min, Max] | [0.600, 552] | [63.0, 705] | [0.600, 705] |  |
| **LA volume (mL)** | Mean (SD) | 85.0 (29.9) | 83.7 (29.3) | 84.6 (29.7) | 0.499 |
|  | Median [Q1, Q3] | 79.0 [64.7, 102] | 81.0 [63.0, 102] | 80.0 [64.0, 102] |  |
|  | [Min, Max] | [8.00, 238] | [10.0, 261] | [8.00, 261] |  |
| **RA area (cm2)** | Mean (SD) | 18.2 (5.25) | 19.6 (5.88) | 18.6 (5.51) | <0.001 |
|  | Median [Q1, Q3] | 17.0 [15.0, 21.0] | 19.0 [15.5, 21.9] | 18.0 [15.0, 21.6] |  |
|  | [Min, Max] | [0, 43.0] | [6.10, 65.0] | [0, 65.0] |  |
| **LV SEPT DIAS (cm)** | Mean (SD) | 1.15 (0.218) | 1.14 (0.197) | 1.15 (0.211) | 0.0689 |
|  | Median [Q1, Q3] | 1.11 [1.00, 1.30] | 1.14 [1.01, 1.20] | 1.13 [1.00, 1.25] |  |
|  | [Min, Max] | [0.500, 2.20] | [0.654, 2.39] | [0.500, 2.39] |  |
| **LV PW DIAS (cm)** | Mean (SD) | 1.04 (0.188) | 1.04 (0.177) | 1.04 (0.184) | 0.448 |
|  | Median [Q1, Q3] | 1.00 [0.900, 1.10] | 1.05 [0.943, 1.10] | 1.02 [0.900, 1.10] |  |
|  | [Min, Max] | [0.500, 2.10] | [0.0820, 2.02] | [0.0820, 2.10] |  |
| **LVEF (%)** | Mean (SD) | 53.9 (13.2) | 51.4 (12.5) | 53.0 (13.0) | <0.001 |
|  | Median [Q1, Q3] | 59.7 [50.0, 60.0] | 52.1 [46.3, 60.0] | 57.0 [46.7, 60.0] |  |
|  | [Min, Max] | [7.00, 84.0] | [8.00, 86.0] | [7.00, 86.0] |  |
| **SV (mL)** | Mean (SD) | 84.8 (22.6) | 70.1 (26.9) | 79.8 (25.1) | <0.001 |
|  | Median [Q1, Q3] | 84.3 [70.0, 97.0] | 67.0 [51.0, 86.0] | 79.8 [62.4, 94.3] |  |
|  | [Min, Max] | [19.2, 185] | [16.0, 293] | [16.0, 293] |  |
| **SVi (mL/m^2^)** | Mean (SD) | 44.2 (11.4) | 37.3 (14.1) | 41.8 (12.8) | <0.001 |
|  | Median [Q1, Q3] | 44.3 [36.7, 50.2] | 35.9 [27.7, 45.1] | 41.9 [33.4, 49.1] |  |
|  | [Min, Max] | [10.3, 96.0] | [9.05, 181] | [9.05, 181] |  |
| **LVEDV (mL)** | Mean (SD) | 113 (35.6) | 109 (40.0) | 112 (37.2) | 0.0239 |
|  | Median [Q1, Q3] | 108 [88.2, 130] | 106 [81.6, 131] | 107 [86.3, 131] |  |
|  | [Min, Max] | [15.6, 359] | [31.1, 290] | [15.6, 359] |  |
| **LV_DIAS_DIM_CM** | Mean (SD) | 4.73 (0.722) | 5.37 (1.23) | 4.95 (0.972) | <0.001 |
|  | Median [Q1, Q3] | 4.70 [4.23, 5.20] | 4.92 [4.52, 6.09] | 4.80 [4.35, 5.30] |  |
|  | [Min, Max] | [2.20, 7.60] | [3.59, 10.2] | [2.20, 10.2] |  |
| **LVMI** (g/m^2^) | Mean (SD) | 102 (24.3) | 113 (29.0) | 106 (26.5) | <0.001 |
|  | Median [Q1, Q3] | 102 [82.4, 117] | 111 [93.8, 129] | 105 [86.0, 121] |  |
|  | [Min, Max] | [43.0, 258] | [43.7, 237] | [43.0, 258] |  |
| **LA Area** (cm^2^) | Mean (SD) | 24.4 (5.84) | 24.3 (6.48) | 24.4 (6.07) | 0.819 |
|  | Median [Q1, Q3] | 24.0 [20.0, 28.0] | 24.0 [20.0, 28.0] | 24.0 [20.0, 28.0] |  |
|  | [Min, Max] | [10.0, 57.7] | [8.00, 54.0] | [8.00, 57.7] |  |
| **Heart failure (baseline)** | 0 | 1749 (79.1%) | 537 (47.1%) | 2286 (68.2%) | <0.001 |
|  | 1 | 463 (20.9%) | 604 (52.9%) | 1067 (31.8%) |  |
| **IHD (baseline)** | 0 | 1818 (82.2%) | 890 (78.0%) | 2708 (80.8%) | 0.0143 |
|  | 1 | 394 (17.8%) | 251 (22.0%) | 645 (19.2%) |  |
| **MI (baseline)** | 0 | 1635 (73.9%) | 651 (57.1%) | 2286 (68.2%) | <0.001 |
|  | 1 | 577 (26.1%) | 490 (42.9%) | 1067 (31.8%) |  |
| **Atherosclerosis, CAD and PVD (baseline)** | 0 | 1621 (73.3%) | 712 (62.4%) | 2333 (69.6%) | <0.001 |
|  | 1 | 591 (26.7%) | 429 (37.6%) | 1020 (30.4%) |  |
| **Rheumatic heart disease (baseline)** | 0 | 2125 (96.1%) | 1090 (95.5%) | 3215 (95.9%) | 0.76 |
|  | 1 | 87 (3.9%) | 51 (4.5%) | 138 (4.1%) |  |
| **Nonrheumatic aortic valve disease (baseline)** | 0 | 1953 (88.3%) | 998 (87.5%) | 2951 (88.0%) | 0.785 |
|  | 1 | 259 (11.7%) | 143 (12.5%) | 402 (12.0%) |  |
| **Nonrheumatic mitral valve disease (baseline)** | 0 | 2177 (98.4%) | 1085 (95.1%) | 3262 (97.3%) | <0.001 |
|  | 1 | 35 (1.6%) | 56 (4.9%) | 91 (2.7%) |  |
| **Conduction disorder (baseline)** | 0 | 2000 (90.4%) | 1012 (88.7%) | 3012 (89.8%) | 0.295 |
|  | 1 | 212 (9.6%) | 129 (11.3%) | 341 (10.2%) |  |
| **Arrhythmias (baseline)** | 0 | 1975 (89.3%) | 867 (76.0%) | 2842 (84.8%) | <0.001 |
|  | 1 | 237 (10.7%) | 274 (24.0%) | 511 (15.2%) |  |
| **Pulmonary hypertension (baseline)** | 0 | 2081 (94.1%) | 997 (87.4%) | 3078 (91.8%) | <0.001 |
|  | 1 | 131 (5.9%) | 144 (12.6%) | 275 (8.2%) |  |
| **CVA (baseline)** | 0 | 2053 (92.8%) | 952 (83.4%) | 3005 (89.6%) | <0.001 |
|  | 1 | 159 (7.2%) | 189 (16.6%) | 348 (10.4%) |  |
| **Hypertension (baseline)** | 0 | 803 (36.3%) | 326 (28.6%) | 1129 (33.7%) | <0.001 |
|  | 1 | 1409 (63.7%) | 815 (71.4%) | 2224 (66.3%) |  |
| **Dialysis (baseline)** | 0 | 1969 (89.0%) | 897 (78.6%) | 2866 (85.5%) | <0.001 |
|  | 1 | 243 (11.0%) | 244 (21.4%) | 487 (14.5%) |  |
| **CKD (baseline)** | 0 | 1748 (79.0%) | 776 (68.0%) | 2524 (75.3%) | <0.001 |
|  | 1 | 464 (21.0%) | 365 (32.0%) | 829 (24.7%) |  |
| **Syncope (baseline)** | 0 | 2047 (92.5%) | 917 (80.4%) | 2964 (88.4%) | <0.001 |
|  | 1 | 165 (7.5%) | 224 (19.6%) | 389 (11.6%) |  |
| **Hypotension (baseline)** | 0 | 1822 (82.4%) | 771 (67.6%) | 2593 (77.3%) | <0.001 |
|  | 1 | 390 (17.6%) | 370 (32.4%) | 760 (22.7%) |  |
| **Diabetes with complications (baseline)** | 0 | 1502 (67.9%) | 615 (53.9%) | 2117 (63.1%) | <0.001 |
|  | 1 | 710 (32.1%) | 526 (46.1%) | 1236 (36.9%) |  |
| **Diabetes without complications (baseline)** | 0 | 1903 (86.0%) | 810 (71.0%) | 2713 (80.9%) | <0.001 |
|  | 1 | 309 (14.0%) | 331 (29.0%) | 640 (19.1%) |  |
| **Hyperlipidemia (baseline)** | 0 | 1885 (85.2%) | 960 (84.1%) | 2845 (84.8%) | 0.711 |
|  | 1 | 327 (14.8%) | 181 (15.9%) | 508 (15.2%) |  |
| **Cancer (baseline)** | 0 | 1847 (83.5%) | 938 (82.2%) | 2785 (83.1%) | 0.641 |
|  | 1 | 365 (16.5%) | 203 (17.8%) | 568 (16.9%) |  |
| **Bicuspid** | 0 | 2020 (91.3%) | 1112 (97.5%) | 3132 (93.4%) | <0.001 |
|  | 1 | 192 (8.7%) | 29 (2.5%) | 221 (6.6%) |  |
| **Calcified** | 0 | 800 (36.2%) | 718 (62.9%) | 1518 (45.3%) | <0.001 |
|  | 1 | 1412 (63.8%) | 423 (37.1%) | 1835 (54.7%) |  |
| **Heart block (baseline)** | 0 | 2179 (98.5%) | 1042 (91.3%) | 3221 (96.1%) | <0.001 |
|  | 1 | 33 (1.5%) | 99 (8.7%) | 132 (3.9%) |  |
| **Sinus bradycardia** | 0 | 2113 (95.5%) | 1015 (89.0%) | 3128 (93.3%) | <0.001 |
|  | 1 | 99 (4.5%) | 126 (11.0%) | 225 (6.7%) |  |
| **Sinus tachycardia** | 0 | 2159 (97.6%) | 941 (82.5%) | 3100 (92.5%) | <0.001 |
|  | 1 | 53 (2.4%) | 200 (17.5%) | 253 (7.5%) |  |
| **AF or atrial flutter (baseline)** | 0 | 1667 (75.4%) | 684 (59.9%) | 2351 (70.1%) | <0.001 |
|  | 1 | 545 (24.6%) | 457 (40.1%) | 1002 (29.9%) |  |
| **Pacemaker (baseline)** | 0 | 2092 (94.6%) | 1040 (91.1%) | 3132 (93.4%) | <0.001 |
|  | 1 | 120 (5.4%) | 101 (8.9%) | 221 (6.6%) |  |
| **Congenital heart disease (baseline)** | 0 | 2190 (99.0%) | 1129 (98.9%) | 3319 (99.0%) | 0.988 |
|  | 1 | 22 (1.0%) | 12 (1.1%) | 34 (1.0%) |  |
| **PE (baseline)** | 0 | 2111 (95.4%) | 1034 (90.6%) | 3145 (93.8%) | <0.001 |
|  | 1 | 101 (4.6%) | 107 (9.4%) | 208 (6.2%) |  |
| **Cardiomyopathy (baseline)** | 0 | 1530 (69.2%) | 1033 (90.5%) | 2563 (76.4%) | <0.001 |
|  | 1 | 682 (30.8%) | 108 (9.5%) | 790 (23.6%) |  |

**Supplementary Table 5.** Baseline characteristics between the internal validation cohorts of the derivation dataset – Internal training vs. internal test datasets.

| **Characteristics** | **Test** | **Train** | **Overall** | **P-value** |
| --- | --- | --- | --- | --- |
|  | **(N=442)** | **(N=1772)** | **(N=2214)** |  |
| **Demographic** | | | | |
| Age (years) | 73.4 (10.9) | 73.4 (11.0) | 73.4 (11.0) | 1 |
| BMI (kg/m^2^) | 28.2 (8.48) | 28.1 (10.1) | 28.1 (9.79) | 0.98 |
| Height (cm) | 169 (9.55) | 169 (9.98) | 169 (9.90) | 0.988 |
| Male, n (%) | 287 (64.9%) | 1167 (65.9%) | 1454 (65.7%) | 0.935 |
| Weight (kg) | 84.3 (19.1) | 83.9 (20.0) | 84.0 (19.8) | 0.924 |
| **Echocardiographic** | | | | |
| AV mean gradient (mmHg) | 19.4 (7.48) | 19.0 (7.42) | 19.1 (7.43) | 0.55 |
| AV V_max_ (cm/s) | 272 (64.9) | 265 (67.7) | 267 (67.2) | 0.239 |
| AV VTI (cm) | 60.1 (16.5) | 58.5 (16.3) | 58.8 (16.4) | 0.172 |
| AVA (cm^2^) | 1.41 (0.291) | 1.43 (0.292) | 1.43 (0.292) | 0.326 |
| AVAi (cm^2^/m^2^) | 0.730 (0.154) | 0.745 (0.151) | 0.742 (0.152) | 0.2 |
| DSI | 0.379 (0.104) | 0.477 (1.31) | 0.458 (1.17) | 0.29 |
| Diastolic BP (mmHg) | 72.8 (8.47) | 73.4 (7.98) | 73.3 (8.08) | 0.352 |
| Systolic BP (mmHg) | 136 (17.2) | 137 (15.8) | 137 (16.1) | 0.494 |
| Dilated RV, n (%) | 383 (86.7%) | 1620 (91.4%) | 2003 (90.5%) | 0.00938 |
| E/A | 1.07 (0.490) | 1.05 (0.444) | 1.05 (0.454) | 0.578 |
| E/e' | 8.65 (6.48) | 9.17 (6.47) | 9.06 (6.47) | 0.33 |
| LA Area (cm^2^) | 24.4 (5.94) | 24.4 (5.81) | 24.4 (5.84) | 0.996 |
| LA volume (mL) | 85.1 (30.6) | 85.0 (29.7) | 85.0 (29.9) | 0.982 |
| LV PW DIAS (cm) | 1.03 (0.181) | 1.04 (0.190) | 1.04 (0.188) | 0.436 |
| LV SEPT DIAS (cm) | 1.14 (0.211) | 1.16 (0.220) | 1.15 (0.218) | 0.275 |
| LVEDD (cm) | 4.69 (0.708) | 4.73 (0.708) | 4.72 (0.708) | 0.614 |
| LVEDV (mL) | 113 (36.3) | 113 (35.4) | 113 (35.6) | 0.545 |
| LVEF (%) | 54.1 (13.1) | 53.9 (13.2) | 53.9 (13.2) | 0.955 |
| LVMI (g/m^2^) | 101 (24.0) | 103 (24.3) | 102 (24.3) | 0.96 |
| LVOT diameter (cm) | 2.24 (0.216) | 2.23 (0.205) | 2.23 (0.207) | 0.531 |
| LVOT VTI (cm) | 21.4 (5.17) | 21.4 (5.28) | 21.4 (5.26) | 0.737 |
| Moderate or Severe AR, n (%) | 14 (3.2%) | 60 (3.4%) | 74 (3.3%) | 0.999 |
| Moderate or Severe MR, n (%) | 38 (8.6%) | 144 (8.1%) | 182 (8.2%) | 0.974 |
| Moderate or Severe MS, n (%) | 3 (0.7%) | 13 (0.7%) | 16 (0.7%) | 0.949 |
| Moderate or Severe TR, n (%) | 35 (7.9%) | 118 (6.7%) | 153 (6.9%) | 0.993 |
| MV deceleration time (msec) | 237 (69.2) | 239 (66.7) | 239 (67.2) | 0.647 |
| MV E wave (cm/s) | 90.0 (33.3) | 89.6 (34.3) | 89.7 (34.1) | 0.899 |
| PV V_max_ (cm/s) | 92.6 (33.4) | 91.5 (36.2) | 91.7 (35.7) | 0.976 |
| RA area (cm^2^) | 18.2 (5.37) | 18.2 (5.22) | 18.2 (5.25) | 0.846 |
| RAP (mmHg) | 6.07 (3.40) | 5.91 (3.30) | 5.94 (3.32) | 0.984 |
| RV systolic dysfunction, n (%) | 365 (82.6%) | 1476 (83.3%) | 1841 (83.2%) | 0.66 |
| RVSP (mmHg) | 36.2 (10.3) | 35.7 (10.0) | 35.8 (10.1) | 0.937 |
| S prime (cm/s) | 11.8 (2.57) | 12.0 (2.65) | 11.9 (2.63) | 0.615 |
| SV (mL) | 85.5 (22.1) | 84.7 (22.8) | 84.8 (22.6) | 0.387 |
| SVi (mL/m^2^) | 44.4 (11.1) | 44.2 (11.5) | 44.2 (11.4) | 0.782 |
| **Comorbidities** | | | | |
| AF or atrial flutter, n (%) | 105 (23.8%) | 440 (24.8%) | 545 (24.6%) | 0.896 |
| Arrhythmias, n (%) | 38 (8.6%) | 199 (11.2%) | 237 (10.7%) | 0.277 |
| Atherosclerosis, CAD and PVD, n (%) | 116 (26.2%) | 475 (26.8%) | 591 (26.7%) | 0.972 |
| Bicuspid, n (%) | 47 (10.6%) | 145 (8.2%) | 192 (8.7%) | 0.262 |
| Calcified, n (%) | 290 (65.6%) | 1124 (63.4%) | 1414 (63.9%) | 0.695 |
| Cancer, n (%) | 83 (18.8%) | 282 (15.9%) | 365 (16.5%) | 0.349 |
| Cardiomyopathy, n (%) | 126 (28.5%) | 556 (31.4%) | 682 (30.8%) | 0.505 |
| CKD, n (%) | 81 (18.3%) | 383 (21.6%) | 464 (21.0%) | 0.315 |
| Conduction disorder, n (%) | 32 (7.2%) | 180 (10.2%) | 212 (9.6%) | 0.176 |
| Congenital heart disease, n (%) | 5 (1.1%) | 17 (1.0%) | 22 (1.0%) | 0.948 |
| CVA, n (%) | 27 (6.1%) | 132 (7.4%) | 159 (7.2%) | 0.621 |
| Diabetes with complications, n (%) | 143 (32.4%) | 567 (32.0%) | 710 (32.1%) | 0.99 |
| Diabetes without complications, n (%) | 65 (14.7%) | 244 (13.8%) | 309 (14.0%) | 0.879 |
| Dialysis, n (%) | 45 (10.2%) | 198 (11.2%) | 243 (11.0%) | 0.837 |
| Heart block, n (%) | 5 (1.1%) | 28 (1.6%) | 33 (1.5%) | 0.784 |
| Heart failure, n (%) | 99 (22.4%) | 364 (20.5%) | 463 (20.9%) | 0.692 |
| Hyperlipidemia, n (%) | 60 (13.6%) | 267 (15.1%) | 327 (14.8%) | 0.731 |
| Hypertension, n (%) | 285 (64.5%) | 1126 (63.5%) | 1411 (63.7%) | 0.935 |
| Hypotension, n (%) | 81 (18.3%) | 309 (17.4%) | 390 (17.6%) | 0.908 |
| IHD, n (%) | 74 (16.7%) | 320 (18.1%) | 394 (17.8%) | 0.811 |
| MI, n (%) | 118 (26.7%) | 459 (25.9%) | 577 (26.1%) | 0.944 |
| Nonrheumatic aortic valve disease, n (%) | 60 (13.6%) | 199 (11.2%) | 259 (11.7%) | 0.39 |
| Nonrheumatic mitral valve disease, n (%) | 8 (1.8%) | 27 (1.5%) | 35 (1.6%) | 0.911 |
| Obesity, n (%) | 262 (59.3%) | 1109 (62.6%) | 1371 (61.9%) | 0.44 |
| Pacemaker, n (%) | 21 (4.8%) | 99 (5.6%) | 120 (5.4%) | 0.786 |
| PE, n (%) | 16 (3.6%) | 85 (4.8%) | 101 (4.6%) | 0.57 |
| Pulmonary hypertension, n (%) | 22 (5.0%) | 109 (6.2%) | 131 (5.9%) | 0.645 |
| Rheumatic heart disease, n (%) | 21 (4.8%) | 66 (3.7%) | 87 (3.9%) | 0.61 |
| Sinus bradycardia, n (%) | 26 (5.9%) | 75 (4.2%) | 101 (4.6%) | 0.331 |
| Sinus tachycardia, n (%) | 8 (1.8%) | 45 (2.5%) | 53 (2.4%) | 0.668 |
| Syncope, n (%) | 33 (7.5%) | 132 (7.4%) | 165 (7.5%) | 1 |

Continuous variables reported as mean (standard deviation). Categorical variables reported as frequency (proportion). AF, atrial fibrillation; AR, aortic regurgitation; AV, aortic valve; AVA, aortic valve area; AVAi, indexed aortic valve area; BMI, body mass index; BP, blood pressure; CAD, coronary artery disease; CKD, chronic kidney disease; CVA, cerebrovascular accident; DSI, dimensionless severity index; IHD, ischemic heart disease; LA, left atrium; LVEDD, left ventricular end diastolic diameter; LVEDV, left ventricle end-diastolic volume; LVEF, left ventricular ejection fraction; LVOT, left ventricular outflow tract; LV PW, end-diastolic left ventricular posterior wall thickness; LV SEPT DIAS, LV septal end diastole; MI, myocardial infarction; MPG, aortic valve mean pressure gradient; MR, mitral regurgitation; MV, mitral valve; MS, mitral stenosis; NSTEMI, Non-ST elevation myocardial infarction; PE, pulmonary embolism; PVD, peripheral vascular disease; RAP, right atrial pressure; RV, right ventricle; RVSP, right ventricular systolic pressure; STEMI, ST elevation myocardial infarction; SV, stroke volume; SVi, stroke volume index; TR, tricuspid regurgitation; PV, pulmonary valve; RA, right atrium; UA, unstable angina; V_max_, maximum velocity; VTI, velocity time integral.

**Supplementary Table 6. Implications of risk score tertiles.**

**6A -** Survival rates for each risk score tertile in the derivation or external validation cohorts for composite outcome of cardiac death or heart failure hospitalization at 5 years.

| **Risk Score Tertile** | **Risk Score** | **Internal Cohort** | | | **External Validation Cohort** | | |
| --- | --- | --- | --- | --- | --- | --- | --- |
|  |  | **1-year survival, % (95% CI)** | **3-year survival, % (95% CI)** | **5-year survival, % (95% CI)** | **1-year survival, % (95% CI)** | **3-year survival, % (95% CI)** | **5-year survival, % (95% CI)** |
| **Low** | $\text{≤}$3.3 | 95% (94%, 97%) | 90% (88%, 93%) | 84% (81%, 87%) | 93% (90%, 97%) | 83% (79%, 89%) | 77% (71%, 83%) |
| **Intermediate** | >3.3 to 3.9 | 91% (89%, 93%) | 80% (77%, 83%) | 72% (68%, 76%) | 80% (75%, 86%) | 61% (55%, 68%) | 51% (45%, 59%) |
| **High** | >3.9 | 80% (77%, 83%) | 61% (57%, 65%) | 49% (45%, 53%) | 47% (43%, 52%) | 25% (22%, 30%) | 18% (15%, 22%) |

CI, confidence interval.

**6B –**Hazard ratios for each risk score tertile in the derivation or external validation cohorts for composite outcome of 5-year cardiac death or heart failure hospitalization *with* top 10 variables after lasso and greedy selection.

| **Risk Score Tertile** | **Risk Score** | **Internal Cohort** | | | | **External Validation Cohort** | | |
| --- | --- | --- | --- | --- | --- | --- | --- | --- |
|  |  | **HR** | **95% CI** | **p-value** | **HR** | | **95% CI** | **p-value** |
| **Low** | $\text{≤}$3.3 | Reference | | | Reference | | | |
| **Intermediate** | >3.3 to 3.9 | 1.94 | 1.52, 2.48 | <0.001 | 2.62 | | 1.85, 3.70 | <0.001 |
| **High** | >3.9 | 4.27 | 3.41, 5.34 | <0.001 | 7.27 | | 5.37, 9.85 | <0.001 |

CI, confidence interval; HR, hazard ratio.

**6C.** Hazard ratios for each risk score tertile for secondary outcomes and competing risk analyses.

| **Risk Score Tertile** | **Risk Score** | **Internal Cohort** | | | | **External Validation Cohort** | | |
| --- | --- | --- | --- | --- | --- | --- | --- | --- |
|  |  | **HR** | **95% CI** | **p-value** | **HR** | | **95% CI** | **p-value** |
| **5-year all-cause death** | | | | | | | | |
| **Low** | $\text{≤}$3.3 | — | — |  | — | | — |  |
| **Intermediate** | >3.3 to 3.9 | 2.15 | 1.73, 2.68 | **<0.001** | 2.54 | | 1.84, 3.49 | **<0.001** |
| **High** | >3.9 | 3.49 | 2.83, 4.30 | **<0.001** | 4.07 | | 3.07, 5.41 | **<0.001** |
| **5-year cardiac death** | | | | | | | | |
| **Low** | $\text{≤}$3.3 | — | — |  | — | | — |  |
| **Intermediate** | >3.3 to 3.9 | 2.75 | 1.81, 4.18 | **<0.001** | 5.63 | | 2.50, 12.7 | **<0.001** |
| **High** | >3.9 | 7.30 | 4.97, 10.7 | **<0.001** | 16.4 | | 7.71, 34.8 | **<0.001** |
| **5-year cardiac death (death from other causes as competing risk)** | | | | | | | | |
| **Low** | $\text{≤}$3.3 | — | — |  | — | | — |  |
| **Intermediate** | >3.3 to 3.9 | 2.47 | 1.54, 3.96 | **<0.001** | 4.48 | | 1.98, 10.1 | **<0.001** |
| **High** | >3.9 | 7.34 | 4.79, 11.2 | **<0.001** | 12.1 | | 5.72, 25.7 | **<0.001** |
| **5-year heart failure hospitalization** | | | | | | | | |
| **Low** | $\text{≤}$3.3 | — | — |  | — | | — |  |
| **Intermediate** | >3.3 to 3.9 | 1.80 | 1.41, 2.29 | **<0.001** | 2.07 | | 1.50, 2.86 | **<0.001** |
| **High** | >3.9 | 2.69 | 2.14, 3.38 | **<0.001** | 4.66 | | 3.53, 6.15 | **<0.001** |
| **5-year heart failure hospitalization (all-cause death as competing risk)** | | | | | | | | |
| **Low** | $\text{≤}$3.3 | — | — |  | — | | — |  |
| **Intermediate** | >3.3 to 3.9 | 2.26 | 1.78, 2.86 | **<0.001** | 1.93 | | 1.41, 2.64 | **<0.001** |
| **High** | >3.9 | 5.07 | 4.09, 6.29 | **<0.001** | 4.28 | | 3.27, 5.60 | **<0.001** |

CI, confidence interval; HR, hazard ratio.

**Supplementary** **Table 7.** Comparing characteristics and proportion of outcomes between the risk score groups in the derivation and external validation cohorts.

| **Characteristics and Outcomes** | **Internal Derivation Cohort** | | | | **External Validation Cohort** | | | |  |
| --- | --- | --- | --- | --- | --- | --- | --- | --- | --- |
|  | **Low Risk (**$\text{≤}$**3.3)** | **Intermediate (>3.3 to 3.9)** | **High (>3.9)** | **P-value** | **Low Risk (**$\text{≤}$**3.3)** | **Intermediate (>3.3 to 3.9)** | **High (>3.9)** | **P-value** |  |
|  | **(N=730)** | **(N=730)** | **(N=752)** |  | **(N=255)** | **(N=264)** | **(N=622)** |  |  |
| Score | 2.94 (0.278) | 3.60 (0.170) | 4.43 (0.421) | <0.001 | 2.81 (0.418) | 3.63 (0.169) | 4.61 (0.502) | <0.001 |  |
| **Demographics** | | | | | | | | | |
| Age (years) | 68.0 (11.3) | 74.5 (9.67) | 77.7 (9.61) | <0.001 | 61.5 (16.5) | 74.0 (10.5) | 78.9 (9.37) | <0.001 |  |
| BMI (kg/m^2^) | 28.7 (9.03) | 28.0 (8.86) | 27.7 (11.3) | 0.307 | 27.8 (11.5) | 30.1 (22.7) | 28.8 (22.6) | 0.633 |  |
| Height (cm) | 168 (10.2) | 169 (9.51) | 169 (9.90) | 0.046 | 163 (10.5) | 164 (12.3) | 163 (12.3) | 0.727 |  |
| Male, n (%) | 437 (59.9%) | 505 (69.2%) | 512 (68.1%) | <0.001 | 107 (42.0%) | 136 (51.5%) | 342 (55.0%) | 0.00647 |  |
| Weight (kg) | 82.9 (20.2) | 84.7 (18.6) | 84.4 (20.5) | 0.31 | 80.5 (21.9) | 82.4 (19.8) | 79.1 (20.2) | 0.178 |  |
| **Echocardiographic parameters** | | | | | | | | | |
| AV mean gradient (mmHg) | 19.8 (7.44) | 18.9 (7.32) | 18.4 (7.47) | 0.00365 | 13.9 (8.03) | 15.0 (8.06) | 12.5 (8.58) | <0.001 |  |
| AV V_max_ (cm/s) | 272 (69.8) | 267 (65.3) | 261 (65.8) | 0.00993 | 237 (72.4) | 248 (68.7) | 220 (74.8) | <0.001 |  |
| AV VTI (cm) | 60.9 (16.2) | 58.8 (15.7) | 56.7 (16.9) | <0.001 | 49.9 (17.3) | 52.0 (17.4) | 45.8 (19.0) | <0.001 |  |
| AVA (cm^2^) | 1.46 (0.309) | 1.44 (0.280) | 1.39 (0.283) | <0.001 | 1.49 (0.309) | 1.44 (0.316) | 1.36 (0.313) | <0.001 |  |
| AVAi (cm^2^/m^2^) | 0.767 (0.165) | 0.741 (0.144) | 0.719 (0.143) | <0.001 | 0.800 (0.192) | 0.756 (0.159) | 0.734 (0.184) | <0.001 |  |
| DSI | 0.498 (1.46) | 0.446 (0.909) | 0.430 (1.08) | 0.721 | 0.538 (0.186) | 0.458 (0.126) | 0.435 (0.119) | <0.001 |  |
| Diastolic BP (mmHg) | 75.9 (7.22) | 73.6 (7.79) | 70.5 (8.28) | <0.001 | 75.0 (5.30) | 72.6 (7.20) | 68.7 (7.15) | <0.001 |  |
| Systolic BP (mmHg) | 139 (15.9) | 139 (15.9) | 134 (15.9) | <0.001 | 135 (11.7) | 135 (12.1) | 129 (12.6) | <0.001 |  |
| Dilated RV, n (%) | 32 (4.4%) | 55 (7.5%) | 124 (16.5%) | <0.001 | 7 (2.7%) | 24 (9.1%) | 133 (21.4%) | <0.001 |  |
| E/A | 0.920 (0.316) | 0.961 (0.353) | 1.27 (0.558) | <0.001 | 0.966 (0.397) | 1.02 (0.428) | 1.32 (0.576) | <0.001 |  |
| E/e' | 7.38 (5.47) | 8.93 (6.32) | 10.8 (7.04) | <0.001 | 11.8 (4.03) | 14.3 (5.28) | 16.9 (5.32) | <0.001 |  |
| LA Area (cm^2^) | 69.9 (22.3) | 82.5 (25.9) | 102 (31.3) | <0.001 | 66.3 (24.9) | 77.4 (24.1) | 93.6 (28.9) | <0.001 |  |
| LA volume (mL) | 21.6 (4.74) | 24.1 (5.23) | 27.5 (5.92) | <0.001 | 20.7 (5.48) | 22.8 (5.42) | 26.3 (6.49) | <0.001 |  |
| LV PW DIAS (cm) | 0.964 (0.158) | 1.04 (0.176) | 1.10 (0.202) | <0.001 | 0.965 (0.139) | 1.02 (0.155) | 1.09 (0.186) | <0.001 |  |
| LV SEPT DIAS (cm) | 1.12 (0.207) | 1.15 (0.209) | 1.19 (0.230) | <0.001 | 1.09 (0.166) | 1.13 (0.179) | 1.16 (0.213) | <0.001 |  |
| LVEDD (cm) | 4.47 (0.606) | 4.71 (0.654) | 4.98 (0.761) | <0.001 | 4.47 (0.670) | 4.68 (0.667) | 5.10 (0.802) | <0.001 |  |
| LVEDV (mL) | 102 (30.2) | 112 (32.4) | 124 (39.8) | <0.001 | 94.1 (27.5) | 102 (35.8) | 119 (43.3) | <0.001 |  |
| LVEF (%) | 58.3 (9.90) | 55.5 (11.8) | 48.1 (15.1) | <0.001 | 58.6 (7.57) | 55.3 (10.9) | 46.8 (12.8) | <0.001 |  |
| LVMI (g/m^2^) | 92.5 (21.1) | 102 (22.8) | 112 (24.5) | <0.001 | 97.7 (24.8) | 108 (24.8) | 122 (29.2) | <0.001 |  |
| LVOT diameter (cm) | 2.22 (0.212) | 2.24 (0.200) | 2.23 (0.208) | 0.432 | 2.06 (0.222) | 2.11 (0.213) | 2.08 (0.213) | 0.0984 |  |
| LVOT VTI (cm) | 23.3 (5.13) | 21.5 (4.66) | 19.6 (5.23) | <0.001 | 24.9 (8.77) | 22.0 (5.55) | 18.1 (5.82) | <0.001 |  |
| Moderate or Severe AR, n (%) | 28 (3.8%) | 16 (2.2%) | 30 (4.0%) | 0.211 | 10 (3.9%) | 9 (3.4%) | 22 (3.5%) | 0.991 |  |
| Moderate or Severe MR, n (%) | 3 (0.4%) | 14 (1.9%) | 165 (21.9%) | <0.001 | 7 (2.7%) | 5 (1.9%) | 155 (24.9%) | <0.001 |  |
| Moderate or Severe MS, n (%) | 1 (0.1%) | 2 (0.3%) | 13 (1.7%) | 0.00106 | 3 (1.2%) | 2 (0.8%) | 6 (1.0%) | 0.971 |  |
| Moderate or Severe TR, n (%) | 7 (1.0%) | 28 (3.8%) | 118 (15.7%) | <0.001 | 1 (0.4%) | 15 (5.7%) | 147 (23.6%) | <0.001 |  |
| MV deceleration time (msec) | 248 (62.3) | 245 (66.3) | 223 (70.0) | <0.001 | 240 (58.4) | 234 (61.9) | 206 (63.2) | <0.001 |  |
| MV E wave (cm/s) | 72.9 (20.4) | 85.4 (27.2) | 110 (39.9) | <0.001 | 78.4 (19.3) | 91.8 (23.4) | 107 (30.6) | <0.001 |  |
| PV V_max_ (cm/s) | 92.1 (38.1) | 94.5 (35.2) | 88.7 (33.5) | 0.021 | 97.9 (34.8) | 98.6 (31.8) | 91.5 (31.9) | 0.00663 |  |
| RA area (cm^2^) | 16.1 (3.83) | 17.9 (4.78) | 20.4 (5.96) | <0.001 | 16.1 (3.55) | 18.2 (4.25) | 21.5 (6.41) | <0.001 |  |
| RAP (mmHg) | 4.80 (2.15) | 5.56 (2.91) | 7.40 (4.04) | <0.001 | 4.63 (2.37) | 5.51 (3.28) | 7.99 (4.37) | <0.001 |  |
| RV systolic dysfunction, n (%) | 48 (6.6%) | 96 (13.2%) | 229 (30.5%) | <0.001 | 5 (2.0%) | 21 (8.0%) | 123 (19.8%) | <0.001 |  |
| RVSP (mmHg) | 31.1 (6.62) | 34.6 (8.26) | 41.5 (11.7) | <0.001 | 31.6 (8.45) | 38.0 (12.8) | 43.7 (13.1) | <0.001 |  |
| S prime (cm/s) | 12.4 (2.35) | 12.4 (2.59) | 11.0 (2.68) | <0.001 | 12.0 (1.98) | 11.4 (2.12) | 10.2 (2.08) | <0.001 |  |
| SV (mL) | 90.5 (22.8) | 85.6 (20.3) | 78.5 (23.0) | <0.001 | 83.2 (31.7) | 76.4 (21.8) | 62.1 (23.8) | <0.001 |  |
| SVi (mL/m^2^) | 47.7 (11.5) | 44.3 (10.1) | 40.7 (11.4) | <0.001 | 44.3 (17.0) | 40.1 (11.3) | 33.2 (12.3) | <0.001 |  |
| **Comorbidities, No. (%)** | | | | | | | | | |
| AF or atrial flutter, n (%) | 71 (9.7%) | 155 (21.2%) | 319 (42.4%) | <0.001 | 36 (14.1%) | 83 (31.4%) | 338 (54.3%) | <0.001 |  |
| Arrhythmias, n (%) | 44 (6.0%) | 68 (9.3%) | 125 (16.6%) | <0.001 | 42 (16.5%) | 54 (20.5%) | 178 (28.6%) | <0.001 |  |
| Atherosclerosis, CAD and PVD, n (%) | 99 (13.6%) | 188 (25.8%) | 304 (40.4%) | <0.001 | 44 (17.3%) | 89 (33.7%) | 296 (47.6%) | <0.001 |  |
| Bicuspid, n (%) | 118 (16.2%) | 43 (5.9%) | 31 (4.1%) | <0.001 | 11 (4.3%) | 8 (3.0%) | 10 (1.6%) | 0.128 |  |
| Calcified, n (%) | 425 (58.2%) | 480 (65.8%) | 507 (67.4%) | 0.00156 | 74 (29.0%) | 104 (39.4%) | 245 (39.4%) | 0.0276 |  |
| Cancer, n (%) | 105 (14.4%) | 128 (17.5%) | 132 (17.6%) | 0.315 | 36 (14.1%) | 59 (22.3%) | 108 (17.4%) | 0.103 |  |
| Cardiomyopathy, n (%) | 102 (14.0%) | 209 (28.6%) | 371 (49.3%) | <0.001 | 9 (3.5%) | 11 (4.2%) | 88 (14.1%) | <0.001 |  |
| CKD, n (%) | 93 (12.7%) | 117 (16.0%) | 254 (33.8%) | <0.001 | 28 (11.0%) | 56 (21.2%) | 281 (45.2%) | <0.001 |  |
| Conduction disorder, n (%) | 33 (4.5%) | 66 (9.0%) | 113 (15.0%) | <0.001 | 15 (5.9%) | 21 (8.0%) | 93 (15.0%) | <0.001 |  |
| Congenital heart disease, n (%) | 12 (1.6%) | 6 (0.8%) | 4 (0.5%) | 0.173 | 5 (2.0%) | 2 (0.8%) | 5 (0.8%) | 0.455 |  |
| CVA, n (%) | 46 (6.3%) | 47 (6.4%) | 66 (8.8%) | 0.229 | 29 (11.4%) | 50 (18.9%) | 110 (17.7%) | 0.0852 |  |
| Diabetes with complications, n (%) | 153 (21.0%) | 230 (31.5%) | 327 (43.5%) | <0.001 | 73 (28.6%) | 105 (39.8%) | 348 (55.9%) | <0.001 |  |
| Diabetes without complications, n (%) | 69 (9.5%) | 107 (14.7%) | 133 (17.7%) | <0.001 | 50 (19.6%) | 67 (25.4%) | 214 (34.4%) | <0.001 |  |
| Dialysis, n (%) | 68 (9.3%) | 72 (9.9%) | 103 (13.7%) | 0.0339 | 34 (13.3%) | 58 (22.0%) | 152 (24.4%) | 0.00397 |  |
| Heart block, n (%) | 3 (0.4%) | 20 (2.7%) | 10 (1.3%) | 0.00339 | 14 (5.5%) | 15 (5.7%) | 70 (11.3%) | 0.00944 |  |
| Heart failure, n (%) | 26 (3.6%) | 97 (13.3%) | 340 (45.2%) | <0.001 | 43 (16.9%) | 101 (38.3%) | 460 (74.0%) | <0.001 |  |
| Hyperlipidemia, n (%) | 22 (3.0%) | 100 (13.7%) | 205 (27.3%) | <0.001 | 4 (1.6%) | 24 (9.1%) | 153 (24.6%) | <0.001 |  |
| Hypertension, n (%) | 418 (57.3%) | 458 (62.7%) | 533 (70.9%) | <0.001 | 121 (47.5%) | 177 (67.0%) | 517 (83.1%) | <0.001 |  |
| Hypotension, n (%) | 85 (11.6%) | 115 (15.8%) | 190 (25.3%) | <0.001 | 45 (17.6%) | 67 (25.4%) | 258 (41.5%) | <0.001 |  |
| IHD, n (%) | 51 (7.0%) | 107 (14.7%) | 236 (31.4%) | <0.001 | 12 (4.7%) | 41 (15.5%) | 198 (31.8%) | <0.001 |  |
| MI, n (%) | 41 (5.6%) | 154 (21.1%) | 382 (50.8%) | <0.001 | 31 (12.2%) | 83 (31.4%) | 376 (60.5%) | <0.001 |  |
| Nonrheumatic aortic valve disease, n (%) | 57 (7.8%) | 73 (10.0%) | 129 (17.2%) | <0.001 | 22 (8.6%) | 34 (12.9%) | 87 (14.0%) | 0.189 |  |
| Nonrheumatic mitral valve disease, n (%) | 4 (0.5%) | 9 (1.2%) | 22 (2.9%) | 0.00252 | 5 (2.0%) | 3 (1.1%) | 48 (7.7%) | <0.001 |  |
| Obesity, n (%) | 279 (38.2%) | 279 (38.2%) | 285 (37.9%) | 0.999 | 94 (36.9%) | 120 (45.5%) | 223 (35.9%) | 0.0571 |  |
| Pacemaker, n (%) | 16 (2.2%) | 44 (6.0%) | 60 (8.0%) | <0.001 | 9 (3.5%) | 14 (5.3%) | 78 (12.5%) | <0.001 |  |
| PE, n (%) | 20 (2.7%) | 27 (3.7%) | 54 (7.2%) | <0.001 | 16 (6.3%) | 22 (8.3%) | 69 (11.1%) | 0.146 |  |
| Pulmonary hypertension, n (%) | 38 (5.2%) | 35 (4.8%) | 58 (7.7%) | 0.0833 | 18 (7.1%) | 34 (12.9%) | 92 (14.8%) | 0.0201 |  |
| Rheumatic heart disease, n (%) | 10 (1.4%) | 25 (3.4%) | 52 (6.9%) | <0.001 | 7 (2.7%) | 6 (2.3%) | 38 (6.1%) | 0.0339 |  |
| Sinus bradycardia, n (%) | 36 (4.9%) | 33 (4.5%) | 30 (4.0%) | 0.856 | 89 (34.9%) | 24 (9.1%) | 13 (2.1%) | <0.001 |  |
| Sinus tachycardia, n (%) | 9 (1.2%) | 20 (2.7%) | 24 (3.2%) | 0.0848 | 34 (13.3%) | 42 (15.9%) | 124 (19.9%) | 0.108 |  |
| Syncope, n (%) | 43 (5.9%) | 48 (6.6%) | 74 (9.8%) | 0.0222 | 32 (12.5%) | 57 (21.6%) | 135 (21.7%) | 0.0152 |  |
| **Intervention** | | | | | | | | | |
| 5-yr AV intervention | 85 (11.6%) | 56 (7.7%) | 46 (6.1%) | 0.00145 | 25 (9.8%) | 17 (6.4%) | 51 (8.2%) | 0.58 |  |
| 1-yr AV Intervention | 19 (2.6%) | 17 (2.3%) | 16 (2.1%) | 0.947 | 7 (2.7%) | 8 (3.0%) | 31 (5.0%) | 0.357 |  |
| **Outcomes** | | | | | | | | | |
| 5-yr all-cause death (censor AVI and severe AS) | 107 (14.7%) | 194 (26.6%) | 291 (38.7%) | <0.001 | 43 (16.9%) | 102 (38.6%) | 324 (52.1%) | <0.001 |  |
| 5-yr cardiac death (censor AVI and severe AS) | 24 (3.3%) | 58 (7.9%) | 160 (21.3%) | <0.001 | 7 (2.7%) | 31 (11.7%) | 177 (28.5%) | <0.001 |  |
| 5-yr HF rehospitalization (censor AVI and severe AS) | 102 (14.0%) | 215 (29.5%) | 446 (59.3%) | <0.001 | 56 (22.0%) | 99 (37.5%) | 387 (62.2%) | <0.001 |  |
| 5-yr composite of cardiac death or HF rehospitalization (censor at AVI and severe AS) | 98 (13.4%) | 178 (24.4%) | 338 (44.9%) | <0.001 | 47 (18.4%) | 99 (37.5%) | 426 (68.5%) | <0.001 |  |
| Follow up duration in years (censor at AVI and severe AS) | 3.78 (1.66) | 3.50 (1.78) | 2.85 (1.90) | <0.001 | 3.34 (1.90) | 2.34 (1.91) | 1.29 (1.61) | <0.001 |  |
| **5-yr HF hospitalization (all-cause death as competing risk) (censor at AVI and severe AS)** | | | | | | | | | |
| no event | 555 (76.0%) | 420 (57.5%) | 242 (32.2%) | <0.001 | 173 (67.8%) | 110 (41.7%) | 126 (20.3%) | <0.001 |  |
| heart failure | 102 (14.0%) | 215 (29.5%) | 446 (59.3%) |  | 56 (22.0%) | 99 (37.5%) | 387 (62.2%) |  |  |
| death | 73 (10.0%) | 95 (13.0%) | 64 (8.5%) |  | 26 (10.2%) | 55 (20.8%) | 109 (17.5%) |  |  |
| **5-yr cardiac death (death from other causes as competing risk) (censor at AVI and severe AS)** | | | | | | | | | |
| no event | 623 (85.3%) | 536 (73.4%) | 461 (61.3%) | <0.001 | 212 (83.1%) | 162 (61.4%) | 298 (47.9%) | <0.001 |  |
| cardiac death | 24 (3.3%) | 58 (7.9%) | 160 (21.3%) |  | 7 (2.7%) | 31 (11.7%) | 177 (28.5%) |  |  |
| death from other causes | 83 (11.4%) | 136 (18.6%) | 131 (17.4%) |  | 36 (14.1%) | 71 (26.9%) | 147 (23.6%) |  |  |

Continuous variables reported as mean (standard deviation). Categorical variables reported as frequency (proportion). AF, atrial fibrillation; AR, aortic regurgitation; AV, aortic valve; AVA, aortic valve area; AVAi, indexed aortic valve area; BMI, body mass index; BP, blood pressure; CAD, coronary artery disease; CKD, chronic kidney disease; CVA, cerebrovascular accident; DSI, dimensionless severity index; IHD, ischemic heart disease; LA, left atrium; LVEDD, left ventricular end diastolic diameter; LVEDV, left ventricle end-diastolic volume; LVEF, left ventricular ejection fraction; LVOT, left ventricular outflow tract; LV PW, end-diastolic left ventricular posterior wall thickness; LV SEPT DIAS, LV septal end diastole; MI, myocardial infarction; MPG, aortic valve mean pressure gradient; MR, mitral regurgitation; MV, mitral valve; MS, mitral stenosis; NSTEMI, Non-ST elevation myocardial infarction; PE, pulmonary embolism; PVD, peripheral vascular disease; RAP, right atrial pressure; RV, right ventricle; RVSP, right ventricular systolic pressure; STEMI, ST elevation myocardial infarction; SV, stroke volume; SVi, stroke volume index; TR, tricuspid regurgitation; PV, pulmonary valve; RA, right atrium; UA, unstable angina; V_max_, maximum velocity; VTI, velocity time integral.

**Supplementary Table 8.** Hazard ratios (exponent of coefficients) from multivariate regression model for 5-year composite outcome (cardiac death or heart failure hospitalization) *with* all 17 variables after lasso and greedy selection.

| **Characteristic** | **HR** | **95% CI** | **P value** |
| --- | --- | --- | --- |
| Age (years) | 1.02 | 1.01, 1.03 | <0.001 |
| Aortic valve area (cm^2^) | 0.86 | 0.61, 1.22 | 0.4 |
| Diastolic blood pressure (mmHg) | 0.99 | 0.98, 1.00 | 0.019 |
| E-wave (m/s) | 1.01 | 1.00, 1.01 | <0.001 |
| End-diastolic left ventricular posterior wall thickness (cm) | 3.24 | 2.05, 5.11 | <0.001 |
| Left ventricular end diastolic diameter (cm) | 1.29 | 1.13, 1.49 | <0.001 |
| Left ventricular ejection fraction (%) | 0.99 | 0.98, 1.00 | 0.009 |
| Left ventricular outflow tract velocity time integral (cm) | 0.97 | 0.95, 0.99 | 0.007 |
| Moderate or severe mitral regurgitation | 1.54 | 1.17, 2.03 | 0.002 |
| Moderate or severe tricuspid regurgitation | 1.23 | 0.90, 1.68 | 0.2 |
| Pulmonary valve maximum velocity (cm/s) | 1.00 | 1.00, 1.01 | 0.078 |
| Calcified | 1.20 | 0.98, 1.46 | 0.078 |
| Diabetes with complications | 1.24 | 1.02, 1.51 | 0.033 |
| Dialysis | 1.07 | 0.81, 1.41 | 0.6 |
| Hyperlipidemia | 1.42 | 1.11, 1.80 | 0.005 |
| Myocardial infarction | 1.47 | 1.21, 1.79 | <0.001 |
| Nonrheumatic aortic valve disease | 1.15 | 0.86, 1.52 | 0.3 |

CI, confidence interval; HR, hazard ratio.

**Supplementary Table 9.** Summary of selected published risk scores for patients diagnosed with aortic stenosis.

| **Reference** | **Study design** | **Country** | **Sample size** | **Population** | **Statistical method for risk score** | **Risk score / Variables included** | **AUC (internal) (95 CI)** | **AUC (external)** | **Comments** |
| --- | --- | --- | --- | --- | --- | --- | --- | --- | --- |
|  |  |  |  |  |  |  |  |  |  |
|  |  |  |  |  |  |  |  |  |  |
|  |  |  |  |  |  |  |  |  |  |
|  |  |  |  |  |  |  |  |  |  |
| Gasperi 2023 [47] | Retrospective cohort | Brazil | 802 | AS patients aged > 18 years who underwent AV intervention | Multiple logistic regression with forward stepwise selection process | CABG associated = 3  Renal failure = 2  NYHA III/IV = 2  Age > 70 years = 1  LVEF < 50 = 1 to predict death during preoperative period and throughout hospitalization period. | 0.77 (0.72-0.82) with whole dataset | NA | Followed during hospital visit only.  Did not perform cross validation or bootstrapping.  No external validation dataset was used. |
| Lertsanguansinchai 2023 [48] | Retrospective cohort | Thailand | 182 | adults 65-year-old and above with symptomatic severe  AS and underwent TAVI | Decision tree model with 100 iterations | 6 parameters (height, chronic lung disease, STS score,  preoperative LVEF, age, and preoperative LVOT VTI) to predict 30-day mortality.  7 parameters (Preoperative LVEF, STS score, heart rate,  systolic blood pressure, home oxygen use, serum creatinine level, and  preoperative LVOT Vmax) to predict 1-year mortality. | 30-day mortality: 0.83 (0.63-0.98) with test set (20 of data)  1-year mortality: 0.71 (  0.60-0.81) | NA | Followed up to 1 year.  Performed 10-fold cross validation. No external validation dataset was used. |
| Namasivayam 2022 [49]  ASteRisk score | Retrospective cohort | United States and Canada | 1,130 for derivation  540 for validation | Moderate AS (AVA < 1.5 cm^2^ and MPG > 20 mmHg) | Bootstrap lasso regression with 100 bootstrap splits | 9 parameters (energy loss, AVA, transvalvular flow rate, MPG, hyperlipidaemia, logical OR (MI, PVD, wall motion abnormality), posterior wall thickness, CKD, CHF) to predict composite of all-cause mortality or AV intervention | 0.74 (0.73-0.76) | 0.78 (0.77-0.80) | Data derived was from follow up between 2006 and 2017. While validation data consist of patients included historical group from 1999 to 2007. |
| Yousef 2022 [50] | Retrospective cohort | United States | 4,203 | Moderate or severe AS (Severe: AVA<1 cm^2^, or DSI< 0.25, or V_max_ >4 m/s,  or MPG >40 mmHg; moderate: AVA 1–1.5 cm^2^, or DSI 0.25–0.5, or  V_max_ 3–4 m/s, or MPG 20–40 mmHg) | Cox proportional regression model | 13 parameters (Age, gender, race/ethnicity, diabetes mellitus, dyslipidaemia, obesity, CKD, stroke, COPD, heart failure, hypertension, AS severity, intervention) to predict 1-year and 5-year mortality | 0.75 for 1-year mortality  0.72 for 5-year mortality | NA | No external validation dataset used. Included most severe values for AS. Unclear if echocardiographic data were validated internally. Bootstrapping or cross-validation was not used. |
| Minamino-Muta 2020 [51] | Retrospective multicentre cohort | Japan | 1,274 | Asymptomatic severe AS (V_max_>4 m/s, MPG>40 mmHg or AVA<1 cm^2^, who were managed conservatively without AVR. | Multivariate logistic regression model | LVEF <60, haemoglobin < 11 g/dL, chronic lung disease (2 points), diabetes mellitus, haemodialysis, and any concomitant valve disease (1 point) | Derivation set (n=849): 0.79 (0.73-0.84) for 1-year of AS related events (HF hospitalization or AS-related death);  Internal Validation set (n=425): 0.76 (0.67-0.83) | NA | Included 27 centres in Japan  Random split in data for derivation and validation sets, did not use external validation set. |
| Saji 2019 [52] | Retrospective, multicentre cohort | Japan | 682 | Severe AS who underwent TAVR: presence of symptoms, degenerative AS, MPG >40 mmHg or V_max_ > 4 m/s, AVA<1 cm^2^, or AVAi<0.6 cm^2^/m^2^ | Multivariate Cox regression with 4 different scores: (1) STS/ACC TAVR risk score based on STS/ACC TVT Registry risk-adjusted mortality model; (2) Logistic EuroScore; (3) EuroScore II; and (4) STS Score | TAVR risk score: Age, sex, race, renal function, presence of lung disease, degree of heart failure, procedural access and acuity status  + Serum albumin + BMI  Evaluated all-cause death following TAVR and complications at 30 days (all-cause death, stroke, life-threatening bleeding, AKI stage 2 or 3, coronary obstruction requiring intervention, major vascular complications, valve-related dysfunction | TAVR risk score + Serum albumin + BMI: 0.79 (0.71-0.88) for all-cause death | At 1 year:  (1) STS/ACC TAVR risk score: 0.69 (0.59-0.79); (2) Logistic EuroScore: 0.56 (0.45-0.68); (3) EuroScore II: 0.66 (0.55-0.76); and (4) STS Score: 0.63 (0.50-0.75)  At 3 years:  (1) STS/ACC TAVR risk score: 0.66 (0.56-0.76); (2) Logistic EuroScore: 0.62 (0.52-0.72); (3) EuroScore II: 0.62 (0.52-0.72); and (4) STS Score: 0.68 (0.59-0.78) | Patients from 3 centres between 2010 and 2018.  This study found additional variables improved discrimination from TAVR risk score alone.  The new risk score with additional parameters were not validated with external set. |
| Holme 2012 [53] | Based on the Simvastatin and Ezetimibe in Aortic  Stenosis (SEAS) trial (randomised controlled trial) | Europe (Norway, Denmark, Sweden, Finland, Germany, UK and Ireland) | 1,534 | 45-85 years, asymptomatic mild-to-moderate AS (Doppler measured V_max_ > 2.5 m/s and < 4 m/s, normal LV systolic function. Patients were randomised to combined simvastatin+ezetimibe vs. placebo. | Backward stepwise Cox regression analysis | Centralised prognostic index (CPI) = (age-67.4)*0.078-(female gender-0.36)*0.286+(smoking-0.184)*0.184+(LVMI-101.7) *0.0054+(bilirubin-1.7)*0.037+(heart rate-68.2)*0.016  +(LnCRP-(-1.5))*0.293. P = 1-(0.904)^Exp(CPI)^ | All patients: 0.76  Intervention group (n=772): 0.722 (SE: 0.030)  Placebo group (n=762): 0.793 (SE: 0.027) for all-cause mortality during median 5 years. | NA | Echocardiography at baseline were used only. No external validation done. |
| Monin 2009 [54] | Prospective cohort study | France | 107 | Moderate to severe AS, V_max_ > 3 m/s or AVA < 1.5 cm^2^, absence of symptoms, normal LVEF > 50 without segmental wall motion abnormality, normal sinus rhythm, no more than mild associated cardiac valve lesion and serum creatinine <160 umol/L | Multiple logistic regression model using backward elimination | [V_max_ (m/s) x 2]+(natural log of BNP x 1.5)+1.5 (if female sex) | 0.90 (0.84, 0.96) for death or AVR within 24 months | Validation cohort (n=107 from Belgium): 0.89 (0.84,0.96) | Small sample size with shorter follow-up duration in validation cohort (21 months vs. 24 months). Majority had severe AS (72). |

AS, aortic stenosis; AV, aortic valvular; CABG, coronary artery bypass grafting; CI, confidence interval; LVEF, left ventricular ejection fraction; NYHA, New York Heart Association; TAVI, transcatheter aortic valvular intervention. Note this table of existing AS risk scores is not systematic; therefore, additional risk scores may exist that were not included.

|  |  |
| --- | --- |
|  |  |
|  |  |
|  |  |
|  |  |

**Supplementary Figure 1.** Plots to test Proportional Hazard assumption of the multivariate Cox model in top 10 variables.

| **A**   | **B**  **** |
| --- | --- |

**Supplementary Figure 2. A.** ROC for risk scores in predicting the 5-year composite outcome in the internal test dataset and external validation datasets. **B.** Time varying ROC curves from year 1 to 5 for risk scores in predicting the composite outcome in patients in the external validation dataset using the multivariate Cox model with top 10 variables.

| **A**   | **B**  **** |
| --- | --- |

**Supplementary Figure 3. A.** ROC for risk scores using logistic regression model with 17 variables in predicting the composite outcome. **B.** Time varying ROC curves from year 1 to 5 for risk scores in predicting the composite outcome in patients in the external validation dataset using the multivariate Cox model with 17 variables.

**References**

[47] Gasperi R, Bodanese LC, Guaragna J, et al. Proposed Risk Score in Patients with Aortic Stenosis Submitted to Valve Replacement Surgery. Braz J Cardiovasc Surg. 2023;38:219-26.

[48] Lertsanguansinchai P, Chokesuwattanaskul R, Petchlorlian A, et al. Machine learning-based predictive risk models for 30-day and 1-year mortality in severe aortic stenosis patients undergoing transcatheter aortic valve implantation. Int J Cardiol. 2023;374:20-6.

[49] Namasivayam M, Myers PD, Guttag JV, et al. Predicting outcomes in patients with aortic stenosis using machine learning: the Aortic Stenosis Risk (ASteRisk) score. Open Heart. 2022;9.

[50] Yousef S, Amabile A, Huang H, et al. One and Five-Year Mortality Risk Prediction in Patients with Moderate and Severe Aortic Stenosis. Journal of Clinical Medicine. 2022;11:2949.

[51] Minamino-Muta E, Kato T, Morimoto T, et al. A risk prediction model in asymptomatic patients with severe aortic stenosis: CURRENT-AS risk score. Eur Heart J Qual Care Clin Outcomes. 2020;6:166-74.

[52] Saji M, Tobaru T, Higuchi R, et al. Usefulness of the Transcatheter Aortic Valve Replacement Risk Score to Determine Mid-Term Outcomes. Circulation Journal. 2019;83:1755-61.

[53] Holme I, Pedersen TR, Boman K, et al. A risk score for predicting mortality in patients with asymptomatic mild to moderate aortic stenosis. Heart. 2012;98:377-83.

[54] Monin J-L, Lancellotti P, Monchi M, et al. Risk Score for Predicting Outcome in Patients With Asymptomatic Aortic Stenosis. Circulation. 2009;120:69-75.
